# Phosphatidylinositol transfer protein-1 integrates insulin/IGF-1 and TOR signaling to negatively regulate lifespan and healthspan in *Caenorhabditis elegans*

**DOI:** 10.1186/s12929-026-01246-x

**Published:** 2026-04-27

**Authors:** Yen-Hung Lin, Yun-Hsun Liao, Sin-Bo Liao, Tzu-Yu Lin, Muniesh Muthaiyan Shanmugam, Pei-Jia Hsu, Chang-Shi Chen, Tsiu-Ting Ching, Oliver Ingvar Wagner, Chiou-Hwa Yuh, Horng-Dar Wang

**Affiliations:** 1 Institute of Biotechnology, National Tsing Hua University, Hsinchu, 300044 Taiwan; 2 Institute of Molecular and Cellular Biology, National Tsing Hua University, Hsinchu, 300044 Taiwan; 3 Department of Life Science, National Tsing Hua University, Hsinchu, 300044 Taiwan; 4 Department of Biochemistry and Molecular Biology, College of Medicine, National Cheng Kung University, Tainan, Taiwan; 5 Institute of Biopharmaceutical Sciences, National Yang Ming Chiao Tung University, Taipei, 112304 Taiwan; 6 Institute of Bioinformatics and Structural Biology, National Tsing Hua University, Hsinchu, 300044 Taiwan; 7 Institute of Molecular and Genomic Medicine, National Health Research Institutes, Zhunan, Miaoli 35053 Taiwan; 8 Present Address: A.I. Virtanen Institute for Molecular Sciences, University of Eastern Finland, 70211 Kuopio, Finland

**Keywords:** Pitp-1, Aging, Healthspan, Longevity, TOR signaling, Insulin/igf-1 signaling, *Caenorhabditis elegans*

## Abstract

**Background:**

Phosphatidylinositol transfer protein-1 (*pitp-1*) is involved in the phosphoinositide (PIP) cycle. The role of *pitp-1* in promoting healthy longevity remains unknown. Our previous work showed that the PIP cycle–related genes *diacylglycerol lipase-1* (*dagl-1)* and *diacylglycerol kinase-5* (*dgk-5)* regulate lifespan, as overexpression of *dagl-1* or knockdown of *dgk-5* prolongs lifespan and enhances oxidative stress resistance through target of rapamycin (TOR) signaling. As *pitp-1* is a key component of this pathway, we investigated its role in lifespan regulation and the underlying mechanisms, aiming to clarify whether it represents a critical regulator of healthy longevity and how it coordinates conserved signaling pathways to regulate aging.

**Methods:**

*Caenorhabditis elegans* (*C. elegans*) mutants, RNAi-mediated knockdown, and transgenic overexpression were applied to assess lifespan, motility, and stress resistance. Temporal and tissue-specific RNAi were applied to identify critical time window and tissue for *pitp-1*-mediated lifespan regulation. TOR signaling was measured by phosphorylated S6 kinase (p-S6K) and puromycin incorporation, and transcriptomic analysis identified affected pathways.

**Results:**

*pitp-1* negatively regulated lifespan and healthspan in *C. elegans*. Genetic deletion or RNAi-mediated knockdown of *pitp-1* extended lifespan, attenuated age-related motility decline, and increased oxidative stress resistance. Temporal and spatial analyses revealed that suppression of *pitp-1* in neurons during early adulthood was sufficient to promote healthy longevity. Mechanistically, these beneficial effects upon *pitp-1* reduction were associated with suppression of TOR signaling. Conversely, *pitp-1* overexpression shortened lifespan and impaired healthspan via TOR activation. Moreover, *pitp-1* was transcriptionally repressed by DAF-16 downstream of insulin/IGF-1 signaling (IIS), and contributed to IIS-mediated longevity regulation. Furthermore, *pitp-1* reduction also improved organismal proteostasis, as evidenced by decreased polyglutamine (polyQ) aggregation and enhanced motility in a neuronal proteotoxicity model.

**Conclusions:**

These findings identify *pitp-1* as a novel regulator of healthy aging, suggesting a role in coordinating IIS and TOR signaling and providing new insights into conserved mechanisms of longevity regulation.

**Supplementary Information:**

The online version contains supplementary material available at 10.1186/s12929-026-01246-x.

## Background

Aging is an inevitable biological process with a progressive decline in physiological integrity, ultimately increasing vulnerability to stress and leading to death. It is a major risk factor for diverse aging-related diseases, including cancer, neurodegenerative disorders, metabolic syndromes, and imposes a substantial burden on global healthcare systems [1, 2]. Thus, identifying genes and molecular mechanisms that promote healthy longevity is urgently needed to improve the quality of life in expanding aging populations.

Target of rapamycin (TOR), also known as mechanistic target of rapamycin (mTOR), signaling and insulin/IGF-1 signaling (IIS) are evolutionarily conserved nutrient-sensing pathways in regulation of aging [3–5]. TOR signaling integrates metabolic cues to control protein synthesis, growth, and metabolism [3, 6]. Suppression of TOR activity reduces phosphorylated S6 kinase (p-S6K) levels, lowers protein translation, dampens anabolic signaling, extends lifespan and enhances stress resistance across species [7–9]. Similarly, reduced IIS signaling suppresses the PI3K–PDK–AKT kinase cascade and prevents the phosphorylation of DAF-16/FOXO by phosphorylated AKT (p-AKT), which allows DAF-16/FOXO to translocate into the nucleus and orchestrate a gene expression program that protects against cellular damage, improves stress resistance, maintains homeostasis, and promotes healthy longevity [3]. Notably, these two pathways are functionally interconnected.

IIS-activated p-AKT inhibits the TSC1/2 complex, which functions as a negative regulator of TOR complex 1 (TORC1) by suppressing the small GTPase Rheb, thereby promoting TORC1 activation [4, 10–12]. Conversely, TOR complex 2 (TORC2) primarily regulates AKT activation and functions upstream of this pathway [13]. This crosstalk fine-tunes cellular and physiological responses to metabolic conditions, highlighting a coordinated regulatory network that governs the aging process. Understanding the integration of TOR signaling and IIS signaling provides crucial insights into the molecular logic of aging and highlights promising targets for interventions aimed at extending lifespan and delaying age-associated physiological decline.

Phosphatidylinositol transfer proteins (PITPs) are conserved lipid transfer proteins that mediate the transport of phosphatidylinositol (PI) and phosphatidic acid (PA) between the endoplasmic reticulum and plasma membrane, playing essential roles in the phosphoinositide (PIP) cycle and maintaining phosphatidylinositol 4,5-bisphosphate (PIP_2_) homeostasis [14–16]. PITPs are conserved across species with homologs among *C. elegans*, *Drosophila melanogaster (D. melanogaster),* and mammals [16, 17], and are classified into two evolutionarily conserved classes of PITPs, class I and class II [17]. In *C. elegans*, *pitp-1*, the sole class II PITP, is primarily expressed in sensory neurons and regulates chemotaxis in response to environmental cues [18]. It also facilitates rapid recovery of feeding behavior after hypoxia by limiting diacylglycerol (DAG) availability and suppressing PKC activity in *mod-1*-expressing neurons [19]. In *D. melanogaster*, the class II PITP ortholog *rdgB* is essential for PIP cycle during phototransduction in photoreceptor cells [20]. In mammals, Nir2, homologous to *pitp-1*, binds PA and enhances the MAPK and PI3K/AKT signaling pathways in response to growth factor stimulation [21]. Suppression of Nir2 reduces breast cancer cell migration and metastasis [22]. Despite the roles of PITPs in neuronal signaling and cancer biology, whether *pitp-1* contributes to lifespan regulation remains unknown.

Our previous study demonstrated that overexpression of the DAG lipase gene *dagl-1* in *C. elegans* and its *D. melanogaster* ortholog *inaE,* or knockdown of the DAG kinase gene *dgk-5* in *C. elegans* and its *D. melanogaster* ortholog *rdgA*, extends lifespan and enhances oxidative stress resistance in both species, likely through reduced PA levels and subsequent inhibition of TOR signaling [7]. In addition, the phospholipase C β (PLCβ) homolog *egl-8*, another PIP cycle component, has been shown to regulate lifespan in *C. elegans*, as the null mutant *egl-8(n488)* exhibits extended longevity [23]. Consistent with our previous findings, in which this *D. melanogaster* genetic screen for stress-resistant mutants identified *inaE* as a longevity-associated factor [7], the same screen also identified *rdgB*, the *D. melanogaster* ortholog of *C. elegans pitp-1*, as a candidate associated with stress resistance. These observations prompted us to investigate whether *pitp-1* participates in lifespan and healthspan regulation in *C. elegans*, and to examine its potential role in conserved nutrient-sensing pathways, particularly IIS and TOR signaling.

## Methods

### *C. elegans* strains

*Caenorhabditis elegans* strains were maintained at 20 °C on NGM agar plates seeded with *Escherichia coli* (*E. coli)* OP50 using standard protocols [24]. The following alleles and strains were used in this study: Wild-type Bristol N2, JN1297: *pitp-1(pe1297)*, *pitp-1(tm1500)*, TU3311: *uIs60 [unc-119p::YFP* + *unc-119p::sid-1]*, TU3401: *sid-1(pk3321); uIs69 [pCFJ90 (myo-2p::mCherry)* + *unc-119p::sid-1]*, WM118: *rde-1(ne300); neIs9 [myo-3::HA::RDE-1* + *rol-6(su1006)]*, VP303: *rde-1(ne219); kbIs7 [nhx-2p::rde-1* + *rol-6(su1006)]*, XE1375: *wpIs36 [unc-47p::mCherry] I. wpSi1 [unc-47p::rde-1::SL2::sid-1* + *Cbr-unc-119(* +*)]; eri-1(mg366); rde-1(ne219); lin-15B(n744)*, XE1474: *wpSi6 [dat-1p::rde-1::SL2::sid-1* + *Cbr-unc-119(* +*)]; eri-1(mg366); rde-1(ne219); lin-15B(n744)*, XE1581: *wpSi10 [unc-17p::rde-1::SL2::sid-1* + *Cbr-unc-119(* +*)]; eri-1(mg366); rde-1(ne219); lin-15B(n744)*, XE1582: *wpSi11[eat-4p::rde-1::SL2::sid-1* + *Cbr-unc-119(* +*)]; eri-1(mg366); rde-1(ne219); lin-15B(n744)*, CB1370: *daf-2(e1370)*, CF1038: *daf-16(mu86)*, RB1828: *dgk-5(ok2366)*, VC1383: *dgk-5(gk691)*, RB1206: *rsks-1(ok1255)*, TG38: *aak-2(gt33)*, EU1: *skn-1(zu67)*, JIN1375: *hlh-30(tm1978)*, RB2325: *sens-1(ok3157)*, *sesn-1(tm2872)*, TJ356: *zIs356[daf-16p::daf-16a/b::GFP* + *rol-6(su1006)]*, CF1553: *muIs84 [sod-3p::GFP* + *rol-6(su1006)]*, AM101: *rmIs110 [F25B3.3p::Q40::YFP]*, N2 *[pitp-1p::GFP; myo-2p::mRFP]*, N2 *[pitp-1p::pitp-1::GFP; myo-2p::mRFP]*, N2 *[pitp-1p::pitp-1; myo-2p::mRFP]*. Strains were obtained from *Caenorhabditis* Genetics Center (CGC) and National BioResource Project (NBRP). All mutant strains used in this study were outcrossed with the N2 wild-type strain at least three times prior to analysis. For generation of N2 *[pitp-1p::GFP; myo-2p::mRFP]*, N2 *[pitp-1p::pitp-1::GFP; myo-2p::mRFP]* and N2 *[pitp-1p::pitp-1; myo-2p::mRFP]*, a plasmid DNA mix consisting of 80 ng/µL of *pitp-1* constructs and 20 ng/µL of co-injection marker, [*myo-2p::mRFP*], was microinjected into the gonad of young adult N2 hermaphrodite animals. For generation of N2 [*pitp-1p::ptr-23; myo-2p::tdtomato*], a plasmid DNA mix consisting of 80 ng/µL of [*pitp-1p::ptr-23*] and 20 ng/µL of co-injection marker, [*myo-2p::tdtomato*], was microinjected into the gonad of young adult N2 hermaphrodites animals. Individual F2 progenies were isolated to establish independent lines. Microinjection of N2 worms with co-injection marker, [*myo-2p::mRFP*] or [*myo-2p::tdtomato*], alone did not affect the mean lifespan of wild-type animals when grown on OP50 or HT115(DE3) bacteria (data not shown).

### RNA interference assay

RNAi was performed by bacterial feeding as previously described [7, 9, 25]. *E. coli* HT115(DE3) (CGC, Cat#HT115) transformed with either empty vector (L4440) or plasmid expressing double-stranded RNA for the desired gene were cultured at 37 °C overnight in LB supplemented with 100 µg/ml ampicillin and 100 µg/ml tetracycline. Bacteria were seeded on nematode growth medium (NGM) plates containing 100 µg/ml ampicillin and 1 mM IPTG. The RNAi clones picked from Julie Ahringer’s library (Source BioScience) were confirmed by sequencing using M13 forward primer (5′-TGTAAAACGACGGCCAGT-3′). RNAi clones generated in this study were constructed by inserting the cDNA of genes into the L4440 vector. For whole life RNAi, synchronized L1 larvae were transferred to RNAi plates at 20 °C. For adult only RNAi, worms were transferred from L4440 plates to RNAi plates at late L4 to young adult stage. Similarly, for RNAi from different adult age, worms were transferred from L4440 plates to RNAi plates at desired adult age.

### Lifespan assay

Lifespan assays were performed as previously described [7, 9, 26, 27]. The worms used for lifespan assays were well fed and maintained at 20 °C for at least three generations. All the lifespan assays were performed without 5-fluoro-2′-deoxyuridine (FUdR). Synchronized worms were placed on NGM plates seeded with OP50 at 20 °C. For RNAi conditions, synchronized worms were placed on NGM plates seeded with HT115(DE3) bacteria containing empty vector and were transferred to RNAi plates at desired age. For rapamycin treatment condition, rapamycin was dissolved in dimethyl sulfoxide (DMSO). The rapamycin solution was added into NGM plates with the final concentration of 100 µM rapamycin. The final concentration of DMSO for each plate was adjusted to 0.2%, including the control. All the plates used for treating rapamycin were used within 3 days. Synchronized worms were transferred from control plates to rapamycin plates at late L4 to young adult stage. When worms reached adulthood, worms were transferred to fresh plates with desired bacteria at a density of 25–35 worms per plate and continually transferred to fresh plates every day until egg-laying ceased. After day 8 of adulthood, living worms were scored every 1–2 days and transferred to fresh plates every 3–7 days until all the worms were dead. Worms which did not move and did not respond to gently touched with a platinum picker were scored as dead. Worms which exploded, crawled off plates, bagged or were accidentally killed were censored. Statistical analysis of lifespan data was performed by OASIS 2 [28].

### RNA extraction and quantitative real-time PCR

RNA extraction and qPCR were performed as previously described [7, 9]. Synchronized worms were harvested at the desired stage and total RNA was extracted with REzol (Protech, PT-KP200CT). Worms were lysed by three times freezing and thawing by liquid nitrogen. RNA was purified by adding chloroform (Sigma, C2432) and precipitated by adding isopropanol (VWR, 0918). Extracted RNA was further purified by RQ1 RNase-Free DNase (Promega, # M6101). 1 µg of total RNA added with random primer (Promega, C118A) and M-MLV reverse transcriptase (Promega, M1701) was used for synthesis of cDNA according to the manufacturer's instructions. Quantitative real-time PCR was set up by using *Power* SYBR™ Green PCR master mix (ABI, 4367659) and performed the reactions by ABI StepOnePlus Real-Time PCR System. The relative expression levels were calculated by ΔΔCt which was normalized by the internal control, *act-1*. The *p*-values were calculated by Student’s t-test or One-way ANOVA. qPCR primers used in this study are listed below. *act-1*, F: 5′-CGCCAACACTGTTCTTTCCG-3′; R: 5′-CTTGATCTTCATGGTTGATGGGG-3′. *pitp-1*, F: 5′-GGACAAGGTTCAAGATCGCC-3′; R: 5′-CTCACGGGAAAGAGCAACCA-3′. *Y54F10AR.1*, F: 5′-CATCCAGACTCCACTCC-3′; R: 5′-CGTATGCGCGTAGTTTTCGAC-3′. *Y71G12B.17*, F: 5′-GGTCTCCTATACGCAGTGTCG-3′; R: 5′-CGGACGCAGTGTGTTACTTG-3′. *sod-3*, F: 5′-GGGAGCACGCCTACTACTTG-3′; R: 5′-AGCATTGGCAAATCTCTCGC-3′. *dod-24*, F: 5′-TGTCCAACACAACCTGCATT-3′; R: 5′-TGTGTCCCGAGTAACAACCA-3′.

### Motility and paralysis assay

Motility and paralysis assays were performed using established methods with minor modifications. For motility assays, synchronized worms at the desired stage were transferred from the NGM plates to the M9 buffer, as previously described [29]. Worms were allowed to acclimate for 1 min, and the number of body bends was counted in 30 s under a stereomicroscope, as previously described. A body bend was defined as a change in the direction of the anterior region. Bending rate was calculated as body bend per second.

For paralysis assays, worms at day 14 adulthood were placed on NGM plates and gently touched by platinum-made picker several times. Worms that failed to move away from their original position but still retained head movement or pharyngeal pump were scored as paralyzed worms, following established criteria [30]. Worms that moved away upon stimulation were scored as non-paralyzed, whereas dead worms were excluded from analysis. Statistical analyses were performed using Two-way ANOVA (motility) or One-way ANOVA/unpaired Student’s t-test (paralysis).

### Polyglutamine (PolyQ) toxicity assay

PolyQ toxicity was assessed using the AM101 strain expressing polyQ (Q40)::YFP in neurons, as previously described [9, 31]. Age-synchronized worms were maintained under standard conditions and imaged at the indicated time points. Fluorescent puncta representing polyQ aggregates were visualized using a ZEISS LSM 800 confocal microscope with a 40 × objective; GFP fluorescence quantification was performed with NIS-Elements (Nikon) software. Puncta number and size were measured per animal and averaged across biological replicates. For functional assessment, motility decline associated with polyQ toxicity was evaluated by measuring body bending rates as described above. These analyses were used as indicators of proteostasis status [32, 33].

### Body size measurement

Synchronized worms at the desired stage were transferred from the NGM plates to 2% agarose pad. Images were captured using a CCD camera (Nikon DS-Ri2) attached to a stereoscopic microscope (Nikon SMZ1500). Open Lab ver.2.2.5 software (Improvision) was used to measure the body size of each worm. The *p*-values were calculated by One-way ANOVA.

### Oxidative stress assay

Oxidative stress was performed using juglone as described previously [34]. The oxidative stress assay was conducted at 20 °C. Young adult hermaphrodites were transferred to 160 µM or 240 µM juglone (5-hydroxyl-1,4-naphthoquinone, sigma, 481–39–0) containing NGM plates which were seeded with bacteria without FUdR to induce oxidative stress. The number of dead worms was recorded every 2–6 h until all worms were dead or censored from analysis because of worms exploded, crawled off plates, bagged or accidentally killed. The survival curves were performed by the percentage of death and the *p*-values were calculated by log-rank test. Statistical analysis of lifespan data was performed by OASIS 2 [28].

### Gene Expression Omnibus (GEO) analysis

The GEO is a public database for high-throughput functional genomics data (https://www.ncbi.nlm.nih.gov/geo/). Publicly available datasets (GSE21784, GSE53890, GSE106672, and GSE77109) were obtained from GEO. The microarray data of GSE21784 contained three biological repeats of synchronized populations of *C. elegans* at three points during aging. The microarray data of GSE53890 contained several samples of adult human brain samples from frontal cortical regions at different ages. The microarray data of GSE106672 contained four biological repeats of synchronized N2 (Bristol), *daf-2(e1370)*. The microarray data of GSE77109 contained 2 replicates, each was collected on day 4 of adulthood, fed by HT1115 bacteria. Gene expression was analyzed by GEO2R to compare two or more groups of samples. The *p*-values were calculated by unpaired student t-test.

### Western blot

Western blot was performed as previously described [7]. Synchronized worms were harvested at the desired stage. 300–500 worms per sample were washed three times by M9 buffer and collected into 1.5 mL tubes. After removing supernatants, WCE buffer (20 mM HEPES, pH 7.4, 0.2 M NaCl, 0.5% Triton X-100, 5% glycerol, 1 mM EDTA, 10 mM β-glycerophosphate, 2 mM NA3VO4, 1 mM NaF, 1 mM DTT) with 1 × cocktail protease inhibitor (Roche) and 1 × phosphatase inhibitor (Roche) was added to each sample. Samples were added with 0.5 mm ZrO beads and homogenized by the Bullet Blender. The concentration of extracted supernatants was detected by Bradford protein assay. The quantified proteins were mixed with 6X sample buffer dye (100 mM tris–HCl, pH 6.8, 4% SDS, 0.2% bromophenol blue, 200 mM 2-mercaptoethanol, 20% glycerol, 8 M urea) and denatured at 95 °C. 30 µg proteins were loaded in 10% SDS-PAGE for protein electrophoresis and transferred to nitrocellulose (NC) membrane by Bio-Rad system. The NC membrane was incubated in 5% BSA in 1 × TBST as the blocking buffer. Immunoblotting was performed by incubating with anti-pS6K (Cell Signaling, Billerica, MA, USA, #9209, 1:500 dilution in 5% BSA /1xTBST), anti-p-AKT (Cell Signaling, #9271, 1:1000 dilution in 5% BSA/1xTBST), anti-puromycin (Merck Millipore, #MABE343, 1:5000 in 5% milk/1XTBST), anti-β-actin (GeneTex, GTX109639, 1:10 000 dilution in 5% milk/1xTBST), or anti-GAPDH (Epitomics, #S0011, 1:2000 in 5% milk/1XTBST). The membrane was washed three times with 1xTBST and incubated with the secondary antibody (Peroxidase-conjugated AffiniPure Goat Anti-Rabbit IgG (H + L), Jackson, 111–035–003, 1:10,000 in 5% BSA/1xTBST for phosphorylated proteins or 5% milk/1xTBST for other proteins). After three times washing by 1xTBST, membrane was incubated with chemiluminescent HRP substrate (Millipore, WBKLS0500) and detected chemiluminescent signals by ImageQuant LAS 4000 mini. The protein image was quantified by ImageJ to calculate the fold changes by normalizing each measurement to its control. The *p*-values were calculated by unpaired Student’s t-test or One-way ANOVA.

### Puromycin incorporation assay

To evaluate global protein synthesis in *C. elegans*, we employed a puromycin incorporation assay adapted with modifications from previously published protocols [35]. Synchronized worms were aged to day 5 of adulthood and collected using M9 buffer. Approximately 500 animals were washed twice with M9 and then resuspended in S-basal medium. For puromycin treatment, OP50 bacteria were grown overnight and subsequently concentrated tenfold in S-basal. Worms were incubated in a 1 mL mixture composed of 750 µL S-basal, 200 µL of the concentrated OP50 suspension, and 50 µL of 10 mg/mL puromycin (Sigma, SI-P8833), yielding a final puromycin concentration of 0.5 mg/mL. Worms were then incubated in a mixture at 200 rpm for 4 h at room temperature. Following treatment, worms were washed three times with ice-cold S-basal, chilled on ice, and snap-frozen in liquid nitrogen. Protein lysates were prepared using RIPA buffer, and puromycin-labeled proteins were detected by anti-puromycin via Western blotting as described previously. After blot stripping, β-actin was probed and used as a loading control.

### SOD-3 and DAF-16 reporter assay

The transgenic strains TJ356 and CF1553 were used in this study. Synchronized worms fed with EV or RNAi clones were harvested at the desired stage. Photos were taken with a CCD camera (Nikon DS-Ri2) attached to a stereoscopic microscope (Nikon SMZ1500) with the X-Cite® 120Q excitation light source (excitation at 470 nm and emission at 535 nm). The mean fluorescence intensity was measured by ImageJ software (NIH). The *p*-values were calculated by unpaired student t-test.

### Fluorescence quantification

Fluorescence quantification was performed using NIS-Elements software (Nikon) and ImageJ (NIH). For each image, regions of interest (ROIs) were defined according to the specific reporter, including neuronal regions for polyQ aggregation assays and whole-animal regions for GFP reporters. Background fluorescence was measured from an adjacent non-fluorescent region and subtracted from the ROI signal. For puncta analysis, GFP puncta were automatically detected using identical thresholding parameters across all samples, and puncta number and size were quantified. For fluorescence intensity analysis, corrected mean fluorescence intensity within the ROI was measured. For DAF-16::GFP localization, fluorescence distribution was examined by confocal microscopy, and representative images were used to assess nuclear localization. All measurements were normalized to the average value of the control group. Image acquisition and analysis parameters were kept constant within each experiment. Statistical analyses were performed using GraphPad Prism 8.0.

### RNA-seq

Synchronized worms were harvested on day 3 of adulthood. The extracted RNA samples were DNase treated and assigned RNA Integrity Number (RIN) quality control. RNA samples with RIN > 7.0 were used to perform next generation RNA sequencing (150 bp, paired-end, ~ 20 million reads/sample, ~ 6G total). Gene expression level was measured by transcript abundance. HISAT2 software was used to read alignment and StringTie software was used to assemble RNA-Seq alignments into potential transcripts in this experiment. Differential expression analysis was performed using DESeq2 software. |FoldChange|> 1.5 and q-value < 0.05 were taken as the differentially expressed gene screening standard. The gene ontology (GO) enrichment analysis and the KEGG pathway analysis were performed by DAVID (https://david.ncifcrf.gov/). The RNA-seq raw data can be accessed by the GEO accession number GSE309580.

## Results

### Reduction of *pitp-1 *promotes healthy longevity in *C. elegans*

**Fig. 1:**
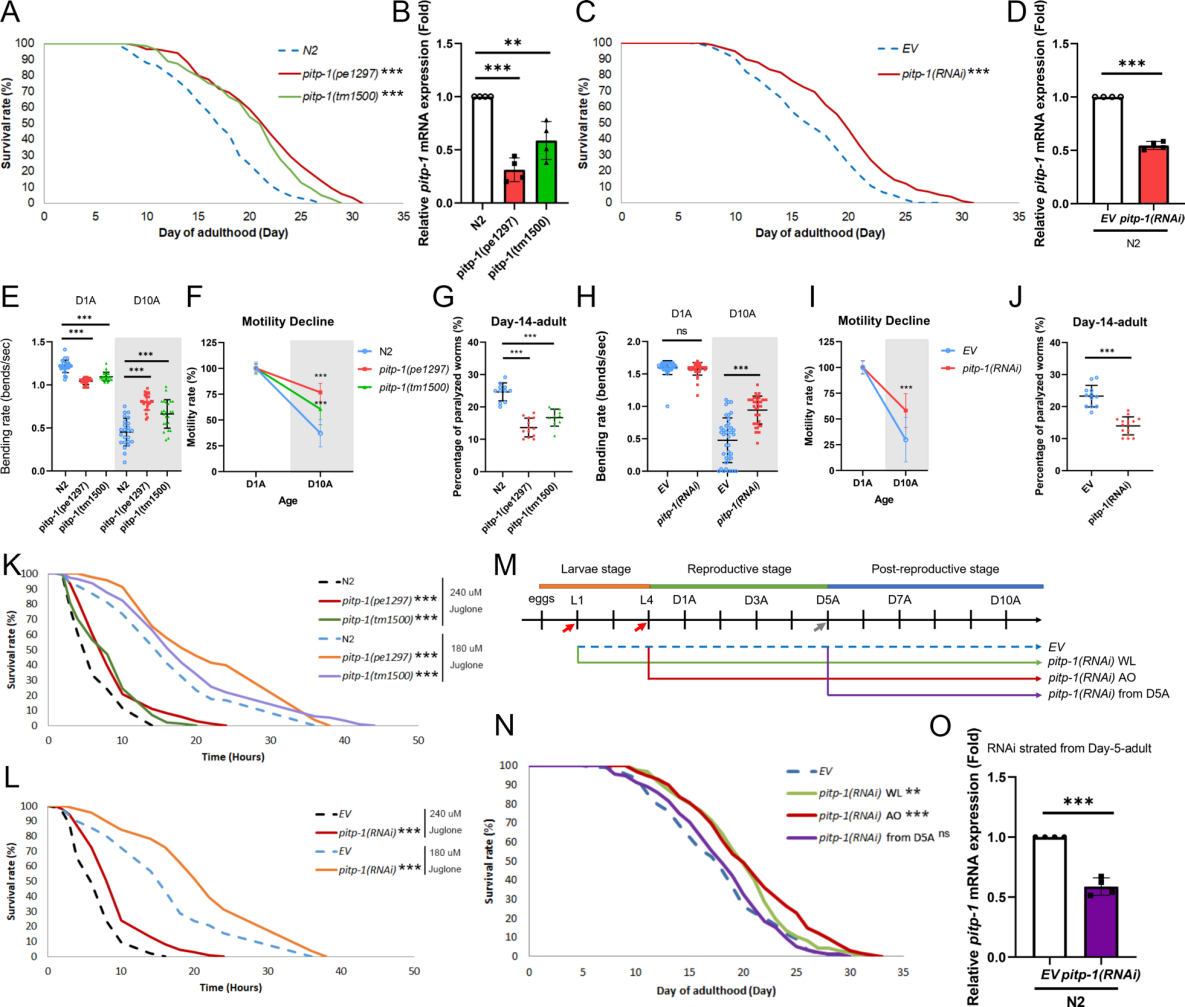
Reduction of *pitp-1* extends lifespan and promotes healthspan. **A** Two independent *pitp-1* mutants displayed significantly extended lifespan. **B** qPCR confirmed reduced *pitp-1* mRNA levels in *pitp-1* mutants. **C** Knockdown of *pitp-1* by RNAi from day-1 adult (D1A) extended lifespan. **D** qPCR confirmed reduced *pitp-1* mRNA expression upon *pitp-1(RNAi)*. **E**, **F** Both *pitp-1* mutants exhibited increased motility and ameliorated motility declines at D10A. **G** Both of the *pitp-1* mutants exhibited less paralyzed worms at D14A. **H**, **I** Knockdown of *pitp-1* displayed enhanced motility and improved motility declines at D10A. **J** Knockdown of *pitp-1* by RNAi ameliorated age-induced paralysis at D14A. **K**, **L** *pitp-1* mutants RNAi-treated worms showed elevated resistance to juglone-induced oxidative stress. **M** Schematic diagram of RNAi treatment timeline. **N** *pitp-1* knockdown during adulthood extended lifespan. **O** qPCR confirmed that *pitp-1*(RNAi) from D5A exhibited lowered *pitp-1* mRNA levels. *p*-values were calculated by log-rank test in (**A**, **C**, **K**, **L**, **N**), and by One-way ANOVA in (**B**, **F**, **G**), and by Two-way ANOVA in (E, H), and by unpaired Student’s t-test in (**D**, **I**, **J**, **O**). Lifespan analysis is representative of at least three independent biological replicates, except oxidative stress assays, which were repeated twice with consistent results. Data are presented as mean ± SD (n = 3 biological replicates) for all quantitative analyses. Statistical significance was determined by log-rank test for lifespan assays, ANOVA for multiple comparisons, and unpaired Student’s t-test where applicable

To examine whether *pitp-1* regulates lifespan in *C. elegans*, we obtained two different *pitp-1* mutants, *pitp-1(pe1297)* and *pitp-1(tm1500)*, for lifespan measurement. The *pitp-1(pe1297)* allele carries a large genomic deletion replaced by a *C. briggsae unc-119(*+*)* cassette and has been described as a candidate null allele with near-complete loss of the PITP domain, whereas the *pitp-1(tm1500)* allele contains a deletion predicted to cause a frameshift and premature truncation, retaining only the N-terminal PITP domain (Supplementary Fig. 1A, 1B) [18]. Both *pitp-1* mutants exhibited significant lifespan extension and lowered *pitp-1* mRNA levels compared to wild-type N2 worms (Fig. 1A, B, and Supplementary Table S1). Similarly, RNAi knockdown of *pitp-1* from day-1 adult (D1A) stage significantly extended lifespan compared to the control worms fed with empty vector (*EV*) (Fig. 1C, D, and Supplementary Table S1). These results indicate that reduction of *pitp-1* expression promotes longevity in *C. elegans*.

*pitp-1* is the single class II PITP gene with a conserved PITP domain in *C. elegans*. Given that the reduction of *pitp-1* extends lifespan, we wondered whether inhibition of the class I PITP genes, *Y54F10AR.1* or *Y71G12B.17*, would result in a similar effect on lifespan. However, knockdown of *Y54F10AR.1* or *Y71G12B.17*, alone or in combination, did not prolong lifespan (Supplementary Fig. 1C and Supplementary Table S1). qPCR confirmed that *Y54F10AR.1* knockdown specifically reduced its own mRNA levels without affecting *Y71G12B.17* or *pitp-1* expression, and vice versa for *Y71G12B.17* knockdown (Supplementary Fig. 1D–1F). These results suggest that reduced expression of the class II PITP gene *pitp-1*, but not other PITP homologs, plays a key role in promoting longevity in *C. elegans*.

We next examined whether reduction of *pitp-1* confers benefits on healthspan. Aging is associated with motility decline and often culminates in paralysis. Thus, we measured locomotor capacity by quantifying body bending rates at day 1 and day 10 of adulthood (D1A and D10A). While *pitp-1* mutants exhibited slightly reduced bending rates compared to N2 at D1A, these mutants retained markedly higher bending rates than age-matched N2 at D10A (Fig. 1E, F), indicating the mutants do not display early-life hyperactivity but show improved preservation of motility upon aging. RNAi knockdown of *pitp-1* produced similar benefits (Fig. 1H, I). Additionally, we assessed age-associated paralysis at D14A as another indicator of motor function. Paralysis at D14A was significantly reduced in both mutants and RNAi-treated worms (Fig. 1G, J), demonstrating a marked delay in paralysis onset. We next examined oxidative stress tolerance, another longevity-associated phenotype. We challenged both *pitp-1* mutants and RNAi-treated worms with juglone (5-hydroxy-1,4-naphthoquinone), a pro-oxidant compound that induces intracellular oxidative damage, and found both mutants and the RNAi-treated worms exhibited significantly increased survival (Fig. 1K, L, and Supplementary Table S2). Long-lived organisms frequently display smaller body size. Consistent with this notion, both *pitp-1* mutants were significantly smaller than wild-type animals (Supplementary Fig. 1G). Together, these results demonstrate that reduction of *pitp-1* is associated with multiple longevity-associated phenotypes, including improved motility, less paralysis, enhanced oxidative stress resistance, and reduced body size, supporting its role in promoting healthspan.

### Knockdown of *pitp-1* before post-reproductive age is essential for enhanced longevity

The timing of longevity intervention is critical, as different lifespan-regulating pathways show distinct temporal requirements. For example, in *C. elegans*, knockdown of *daf-2* during reproductive adulthood is important to extend lifespan [36]. Similarly, overexpression of *dFOXO* during reproductive adulthood promotes longevity in *D. melanogaster* [37]*.* In addition, the geroprotective benefits of TOR inhibition via long-term rapamycin treatment can be achieved with a short-term exposure to rapamycin during early adulthood in *D. melanogaster* and mice [38]. These data suggest the importance of investigating the precise time window for longevity assurance.

To delineate the temporal requirement for *pitp-1* reduction to extend lifespan, we initiated *pitp-1* RNAi knockdown at different stages: L1, late L4 (adult-only, AO), and D5A (Fig. 1M). Knockdown of *pitp-1* from L1 or AO both significantly extended lifespan similarly, suggesting that *pitp-1* reduction from L1 larval development is dispensable and from AO is sufficient for promoting longevity (Fig. 1N and Supplementary Table S1). In contrast, *pitp-1* RNAi initiated at D5A with effective *pitp-1* mRNA reduction produced only a marginal increase in lifespan without statistical significance (Fig. 1N, O), indicating a temporal restriction for the longevity effect. To further narrow the time window, we treated worms with *pitp-1* RNAi starting from D3A, D4A, D5A, or D7A (Supplementary Fig. 1F, 1G, and Supplementary Table S1). *pitp-1* knockdown from D3A or D4A significantly extended lifespan, whereas *pitp-1* knockdown initiated at D5A or later failed to prolong lifespan (Fig. 1N; Supplementary Fig. 1H, 1I, and Supplementary Table S1). Interestingly, the longevity effect of *pitp-1* knockdown was diminished when RNAi was initiated at D4A, suggesting that there is an optimal time window from L4 to D3A during early reproductive age. A previous microarray analysis revealed that *pitp-1* expression declines with age in *C. elegans* [39], with significantly lower levels at D6A and D15A compared to L4 (Supplementary Fig. 2A). This age-associated *pitp-1* downregulation may explain why *pitp-1* knockdown from post-reproductive age no longer influences lifespan. Interestingly, analysis of human microarray data revealed similar age-dependent expression changes in *pitp-1* human orthologs [40]. Specifically, *PITPNM2* and *PITPNM3* expression was significantly reduced in the prefrontal cortex of extremely old individuals (> 90 years) compared to younger adults (< 40 years) (Supplementary Fig. 2B–2E), whereas *PITPNM1* showed a slight, non-significant decline (Supplementary Fig. 2F–2G). Together, these findings suggest that the longevity effect of *pitp-1* suppression is temporally restricted to a critical window prior to the post-reproductive stage, and that age-related reduction of *pitp-1*/PITPNMs expression may represent a conserved, protective feature of aging.

### The reduction of *pitp-1* in neuron is critical for lifespan extension

**Fig. 2:**
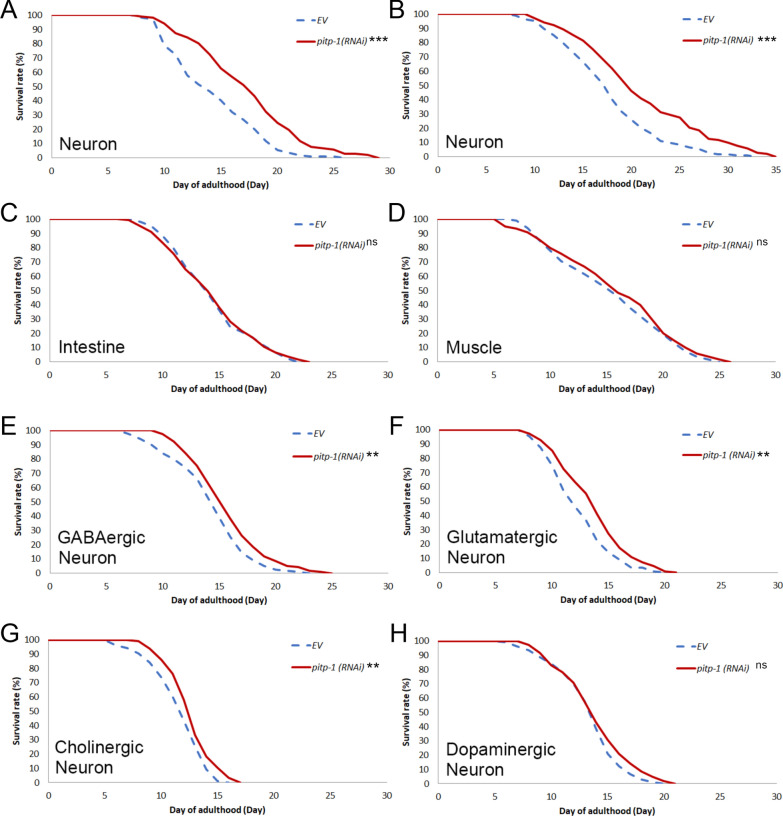
Reduction of *pitp-1* in pan-neuronal tissue and specific neuronal circuits extends lifespan. **A**, **B** Neuron-specific knockdown of *pitp-1* in TU3401 (neuron-restricted RNAi) and TU3311 (whole-body RNAi with enhanced neuronal RNAi) from adulthood increased lifespan. **C**, **D** Intestine-specific or muscle-specific knockdown of *pitp-1* from adulthood did not alter lifespan. **E**–**G** Knockdown of *pitp-1* specifically in GABAergic neuron (XE1375), in glutamatergic neuron (XE1582), or in cholinergic neuron (XE1581) from adulthood extended lifespan. (H) Knockdown of *pitp-1* specifically in dopaminergic neuron (XE1474) showed no significant lifespan increase. Survival curves are representative of at least two independent biological replicates (n = 2–3 depending on the strain). Statistical significance was determined by log-rank test

In addition to the temporal aspect, the spatial effect also plays an important role in longevity. For instance, increased neuronal or intestinal, but not muscular, DAF-16 activity is sufficient for lifespan extension in *C. elegans* [41]. In addition, neuronal TORC1 is essential for TOR-mediated aging regulation [42]. To evaluate the effect of tissue-specific *pitp-1* reduction on longevity, we performed RNAi knockdown in various tissue-specific RNAi strains. Neuronal knockdown of *pitp-1* in TU3401 (neuron-restricted RNAi) and TU3311 (whole-body RNAi with enhanced neuronal RNAi) significantly extended lifespan (Fig. 2A, B and Supplementary Table S3), whereas *pitp-1* knockdown in the intestine or muscle had no effect (Fig. 2C, D and Supplementary Table S3), indicating neuron tissue is critical for *pitp-1*-mediated lifespan regulation. To further investigate which neuron circuits may participate in the longevity upon *pitp-1* reduction, we performed *pitp-1* knockdown in either GABAergic, glutamatergic, cholinergic or dopaminergic neuronal circuit-specific strains individually for the lifespan measurement. Interestingly, except for dopaminergic neurons, knockdown of *pitp-1* in either GABAergic, glutamatergic, or cholinergic neurons is sufficient to extend lifespan (Fig. 2E–H and Supplementary Table S3). These findings indicate that neuronal *pitp-1* suppression, particularly in certain specific neuron types, plays a central role in mediating its longevity effect.

### Overexpression of *pitp-1* decreases lifespan and impairs healthspan

**Fig. 3:**
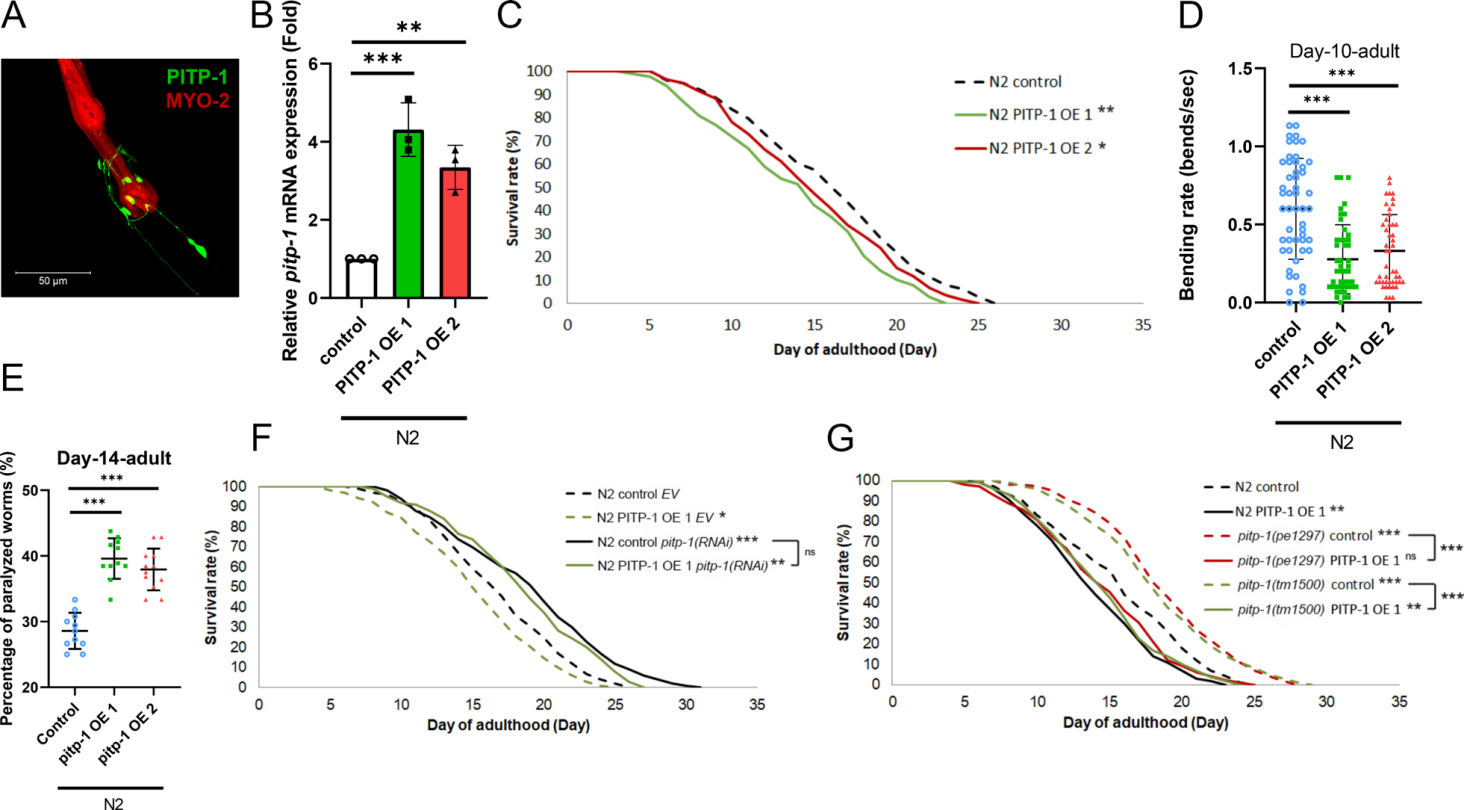
Overexpression of *pitp-1* decreases lifespan and impairs healthspan. **A** The confocal image showed *pitp-1* expression (GFP) in neuron and co-injection marker (mRFP) in PITP-1 OE 1. **B** qPCR confirmed elevated *pitp-1* mRNA levels in the *pitp-1* overexpression strains. **C** Overexpression of *pitp-1* reduced lifespan in both transgenic lines. **D**, **E** PITP-1-overexpressing worms displayed decreased body bending rate at D10A and increased paralysis at D14A compared to control line. **F** The reduced lifespan in N2 PITP-1 OE 1 can be rescued by *pitp-1*(RNAi) knockdown. **G** Overexpression of PITP-1 OE 1 reverted the extended lifespan in both *pitp-1* mutants. Survival curves are representative of three biological replicates. Data are presented as mean ± SD (n = 3 biological replicates) for all quantitative analyses. Statistical significance was determined by log-rank test for lifespan assays, ANOVA for multiple comparisons

To examine whether overexpressing *pitp-1* has the opposite effects on lifespan and healthspan, we generated *pitp-1* overexpressing transgenic worms*,* one line with *pitp-1*::GFP fusion construct under *pitp-1* promoter, N2*[pitp-1p::pitp-1::GFP; myo-2p::mRFP]* (named N2 PITP-1 OE 1) with the control line, N2*[pitp-1p::GFP]* (named N2 control). To exclude possible GFP effects, we also generated *pitp-1* overexpressing without GFP fusion transgenic worms, N2*[pitp-1p::pitp-1; myo-2p::mRFP]* (named N2 PITP-1 OE 2). Confocal imaging confirmed the neuronal expression of the *pitp-1::GFP* fusion protein (Fig. 3A), consistent with the previous reports [18]. Both strains showed about 3-fourfold increase in *pitp-1* mRNA (Fig. 3B). Opposite to *pitp-1* reduction, both N2 PITP-1 OE lines exhibited significantly shortened lifespan (Fig. 3C and Supplementary Table S1), reduced motility by at D10A (Fig. 3D), and increased paralysis at D14A (Fig. 3E), indicating deteriorated aging and health. Importantly, lifespan shortening in N2 PITP-1 OE worms was fully rescued by *pitp-1(RNAi)* (Fig. 3F), and overexpression of *pitp-1* in *pitp-1* mutants abolished their extended lifespan (Fig. 3G and Supplementary Table S4). These findings indicate that *pitp-1* acts as a negative regulator for lifespan and healthspan in *C. elegans*.

### *pitp-1* negatively regulates lifespan through modulating TOR signaling

**Fig. 4:**
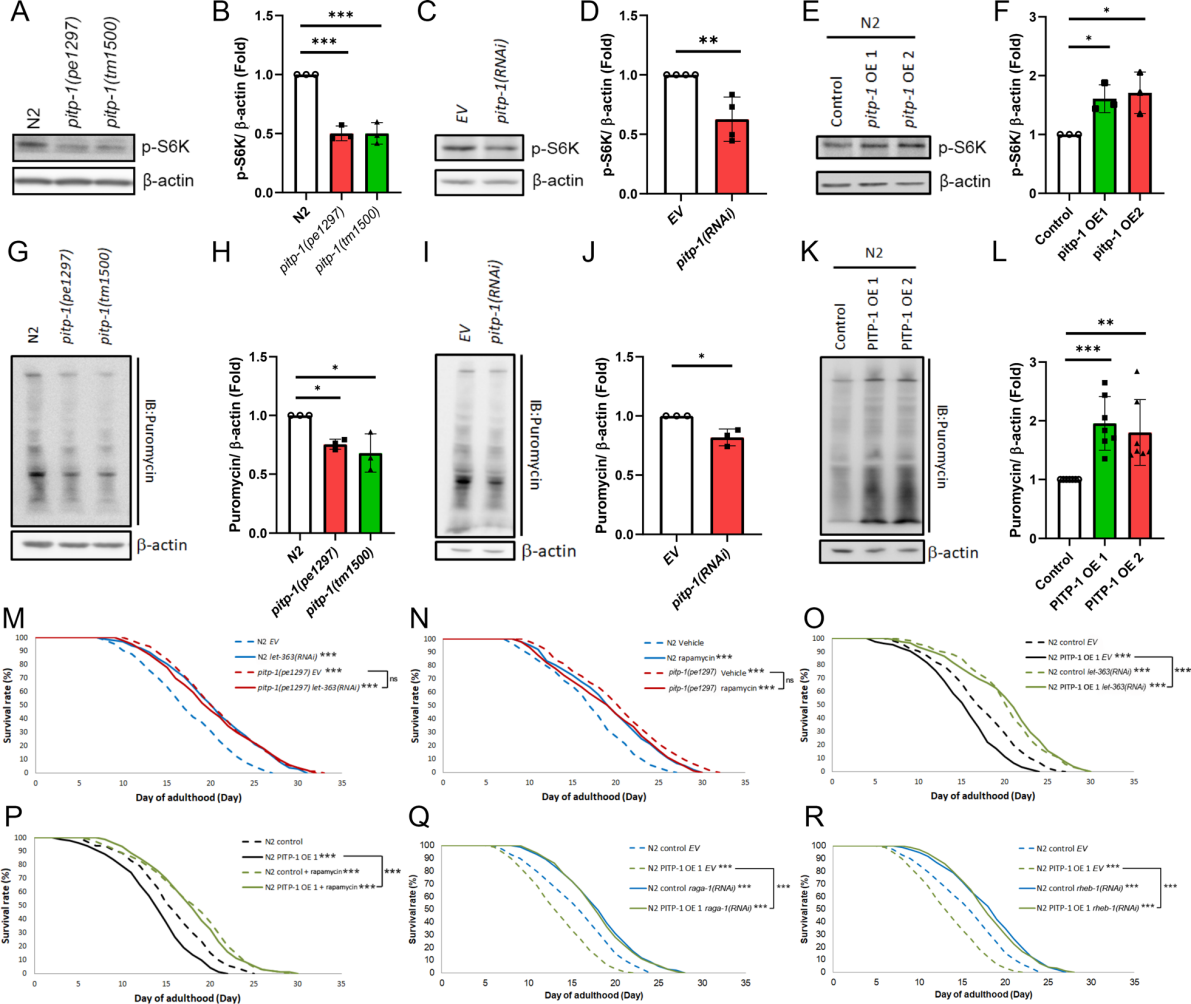
*pitp-1* negatively regulates lifespan through modulating TOR signaling. **A**–**F** *pitp-1* mutants or RNAi knockdown worms exhibited reduced p-S6K levels, whereas PITP-1 overexpression lines displayed increased p-S6K levels. **G**–**L** puromycin incorporation assays showed lowered protein synthesis in *pitp-1* mutants or RNAi knockdown worms, and elevated levels in PITP-1-overexpressing strains. **M**, **N** Genetic or pharmacological inhibition of TOR by *let-363*(RNAi) or rapamycin did not further prolong the longevity of *pitp-1* mutants. **O**, **P** TOR inhibition rescued the shortened lifespan caused by PITP-1 overexpression. **Q**, **R** Knockdown of *raga-1* or *rheb-1*, TOR upstream regulators, rescued the reduced lifespan of PITP-1-overexpressing worms. Survival curves are representative of three independent experiments. Quantitative data are presented as mean ± SD from at least 3 independent experiments. Statistical significance was assessed by log-rank test for lifespan assays, ANOVA for multiple comparisons, and unpaired Student’s t-test where applicable.

Our previous study demonstrated that reduced *dgk-5* extends lifespan through downregulation of TOR signaling [7]. Since *pitp-1* and *dgk-5* function in the same pathway and that reduced expression of either gene leads to longevity, we hypothesized that *pitp-1* may also negatively regulate lifespan via TOR signaling. Supporting this notion, knockdown of *pitp-1* did not further enhance the extended lifespan of *dgk-5* mutants (Supplementary Fig. 3A, 3B and Supplementary Table S4), suggesting *pitp-1* and *dgk-5* act through a common mechanism. Moreover, both *pitp-1* mutants and RNAi-treated worms exhibited significantly reduced p-S6K levels (Fig. 4A–D), indicating diminished TOR signaling. In contrast, overexpression of *pitp-1* markedly increased p-S6K abundance (Fig. 4E and F), suggesting enhanced TOR activation. Furthermore, the elevated TOR signaling in *pitp-1*-overexpressing worms was suppressed by RNAi targeting *let-363*/TOR or its upstream activator *raga-1* (Supplementary Fig. 3C and 3D). Re-expression of *pitp-1* in the *pitp-1* mutant background restored p-S6K levels (Supplementary Fig. 3E, 3F), supporting a role for *pitp-1* as an upstream positive regulator of TOR signaling. Since TOR activation promotes protein translation, we further examined the effect of *pitp-1* on global translation. Both *pitp-1* mutants and RNAi-treated animals showed significantly reduced puromycin labeling (Fig. 4G–4J), indicating decreased protein translation. Conversely, overexpression of *pitp-1* markedly enhanced puromycin incorporation (Fig. 4K, 4L), representing elevated translational output. These results support the notion that *pitp-1* positively regulates TOR activity and downstream protein synthesis. Furthermore, the extended lifespan of *pitp-1* mutants was not further prolonged by either genetic or pharmacological inhibition of TOR (Fig. 4M, 4N, supplementary Fig. 3G, 3H and Supplementary Table S4). Similarly, *pitp-1* RNAi in the *rsks-1/S6K* mutant background failed to further extend the prolonged lifespan (supplementary Fig. 3I and Supplementary Table S4). Moreover, suppression of TOR signaling by either *let-363*(RNAi) or rapamycin treatment rescued the lifespan shortening and reduced motility caused by *pitp-1* overexpression (Fig. 4O, 4P, supplementary Fig. 3J and Supplementary Table S4). Collectively, these results demonstrate that *pitp-1* negatively regulates lifespan through modulation of TOR signaling.

### Several TOR regulators are involved in *pitp-1*-mediated lifespan regulation

TOR activity is modulated by amino acids, growth factors, and energy stress via distinct regulators (Supplementary Fig. 3K). To identify upstream regulators linking *pitp-1* to TOR activity, we performed lifespan epistasis tests. RNAi knockdown of *raga-1* or *rheb-1* rescued the shortened lifespan in *pitp-1*-overexpressing animals (Fig. 4Q, 4R and Supplementary Table S4), suggesting that both Rag and Rheb GTPases are involved in *pitp-1*-mediated lifespan regulation. Conversely, knockdown of *pitp-1* still extended lifespan in the AMPK-deficient strain *aak-2(gt33)* (Supplementary Fig. 3L and Supplementary Table S4), suggesting that *pitp-1* regulates lifespan independent of AMPK. Sestrin, a negative regulator of Rag GTPases, is known to inhibit the amino acid sensing arm of TORC1 and promotes longevity in *C. elegans* [43]. Given the role of Rag GTPases in *pitp-1*-mediated lifespan regulation, we next investigated whether *sestrin* is also required for this effect. Notably, the lifespan extension induced by *pitp-1* knockdown was abolished in *sesn-1* mutant worms (Supplementary Fig. 3M, 3N and Supplementary Table S4), indicating that *sestrin* is required for *pitp-1*-mediated lifespan extension. Together, these findings reveal that the sestrin–Rag GTPase axis and Rheb GTPase, upstream regulators of TOR, are involved in *pitp-1*-mediated lifespan regulation.

### *pitp-1* is involved in insulin/IGF-1 signaling-mediated lifespan regulation

**Fig. 5:**
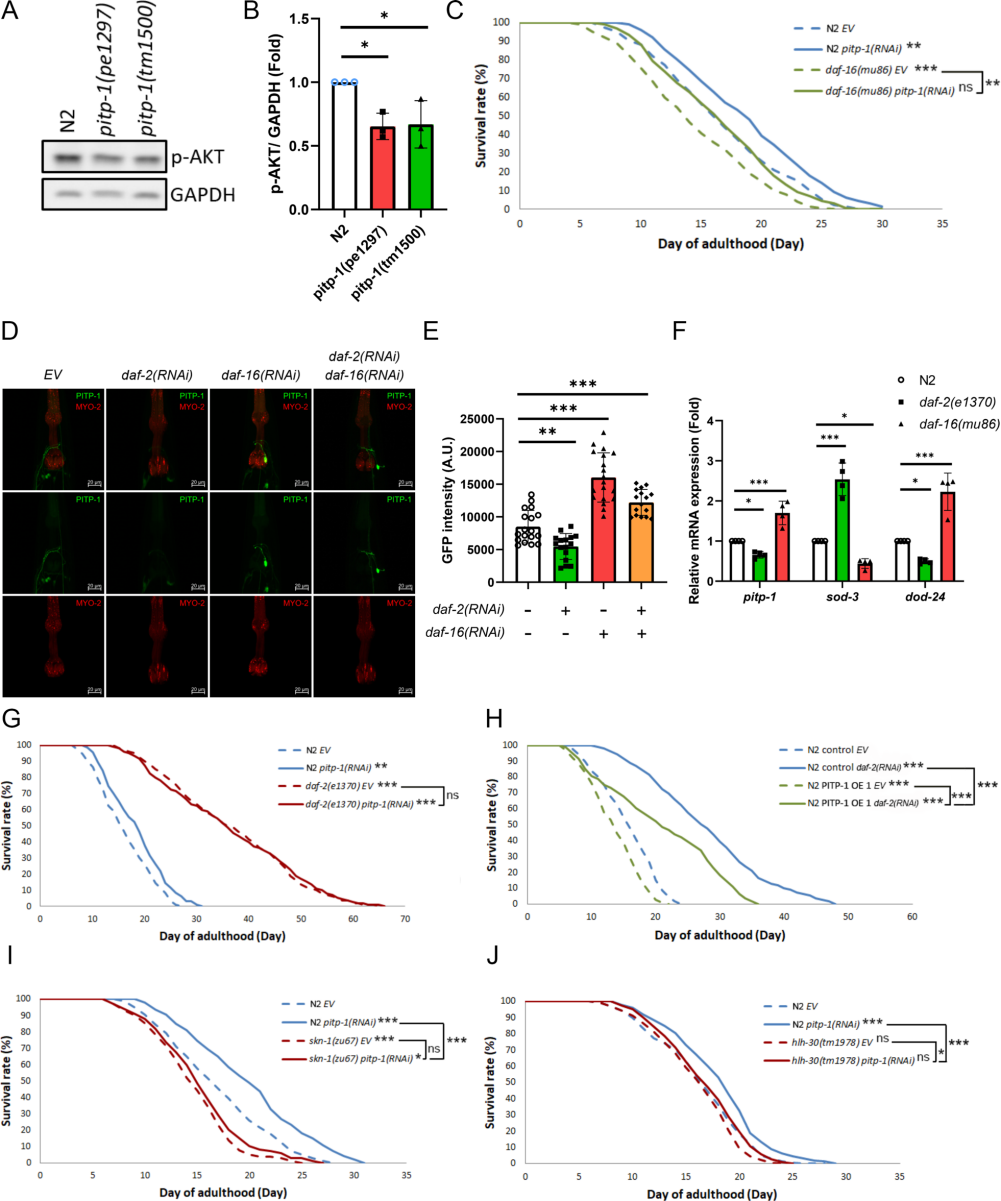
Integration of *pitp-1* in insulin/IGF-1 signaling–mediated lifespan regulation. **A**, **B** *pitp-1* mutants exhibited reduced p-AKT levels compared to N2. **C** Knockdown of *pitp-1* extended lifespan in *daf-16(mu86)*. **D** Representative confocal images of *pitp-1p*::GFP expression following *daf-2* or *daf-16* RNAi. Animals were imaged using a Zeiss LSM800 confocal microscope with a 40× objective. GFP (green) indicates *pitp-1* promoter activity, and MYO-2 (red) serves as a pharyngeal marker. Maximum intensity projections are shown. **E** Quantification of GFP fluorescence intensity. Fluorescence intensity was measured in the defined regions as shown in the middle row of panel D and normalized to the control group by *EV*. **F** Relative mRNA expression levels of *pitp-1* and IIS target genes (*sod-3* and *dod-24*) under indicated conditions. Data are presented as mean ± SD. Statistical significance was determined by One-way ANOVA. **G** Knockdown of *pitp-1* did not further prolong lifespan in *daf-2(e1370)*. **H** PITP-1 overexpression partially blocked the longevity effect by *daf-2(RNAi)* knockdown. **I**, **J** The extended lifespan by *pitp-1* knockdown was abolished in (**I**) *skn-1(zu67)* mutant and diminished in (**J**) *hlh-30(tm1978)* mutant. Survival curves are representative of at least two independent biological replicates (n = 2–3 depending on the strain). Data are presented as mean ± SD. For GFP quantification, n = 10–15 animals were analyzed per condition. For qPCR analyses, n = 4 independent biological samples were used. For immunoblot quantification, n = 3 independent experiments were performed. Statistical significance was determined by log-rank test for lifespan assays, ANOVA for multiple comparisons

Because AKT lies upstream of Rheb-TOR, we first detected the p-AKT levels in long-lived *pitp-1* mutants to check IIS involvement in *pitp-1*-mediated lifespan regulation. Both *pitp-1* mutants exhibit significantly reduced p-AKT levels compared to N2 worms (Fig. 5A and B), suggesting that IIS may be involved in *pitp-1*-mediated lifespan extension. However, knockdown of *pitp-1* did not induce detectable nuclear localization of DAF-16::GFP, nor increase expression of DAF-16 target gene *sod-3* (Supplementary Fig. 4A–4C). These results indicate that *pitp-1* knockdown does not promote DAF-16 transcription activity. To further clarify if DAF-16 is required for *pitp-1* knockdown-mediated lifespan extension, we performed lifespan assays in the *daf-16(mu86)* null mutant by *pitp-1* knockdown. Knockdown of *pitp-1* still extended lifespan in *daf-16(mu86)* mutant (Fig. 5C and Supplementary Table S4), indicating that DAF-16 is not required for the longevity effect by *pitp-1* suppression.

Interestingly, two DAF-16 binding sites were identified in the *pitp-1* promoter [44], raising the possibility that *pitp-1* may act downstream to DAF-16 and be regulated by DAF-16. To test this, we analyzed *pitp-1* promoter-driven GFP expression following RNAi of *daf-2* or *daf-16*. Knockdown of *daf-2* significantly reduced *pitp-1*::GFP expression, while *daf-16* RNAi elevated its expression (Fig. 5D and E). Moreover, co-treatment with *daf-2* and *daf-16* RNAi restored the decreased *pitp-1* GFP intensity caused by *daf-2* knockdown (Fig. 5D and E). Similarly, qPCR confirmed that *pitp-1* transcript levels were downregulated in *daf-2(e1370)* but upregulated in *daf-16(mu86)* mutants (Fig. 5F). Consistently, a previous microarray study also reported reduced *pitp-1* expression in *daf-2(e1370)* (Supplementary Fig. 4D) [45]. These data indicate that *pitp-1* is transcriptionally repressed by DAF-16. Accordingly, *pitp-1* knockdown failed to further extend lifespan in *daf-2(e1370)* mutant (Fig. 5G and Supplementary Table S4), likely due to their already reduced *pitp-1* expression levels. Conversely, overexpression of *pitp-1* partially suppressed the lifespan extension induced by *daf-2(RNAi)* or *age-1(RNAi)* (Fig. 5H, Supplementary Fig. 4E, and Supplementary Table S5), further supporting the notion that *pitp-1* acts as a downstream effector negatively regulated by IIS. Together, these results suggest that *pitp-1* functions downstream of DAF-16 and contributes to IIS-mediated lifespan regulation.

To further clarify how *pitp-1* mediates IIS-dependent but DAF-16-independent lifespan extension, we next examined whether other longevity-associated transcription factors are required for this effect. SKN-1 and HLH-30 are two key regulators of stress response and longevity, and have been implicated in lifespan regulation in response to IIS [46–48]. We therefore performed lifespan assays to determine whether these factors are required for *pitp-1*-mediated longevity. Notably, the longevity induced by *pitp-1* knockdown was abolished in *skn-1(zu67)* mutant, indicating that *skn-1* is required for the longevity effect induced by *pitp-1* reduction (Fig. 5I and Supplementary Table S4). Similarly, the lifespan extension by *pitp-1* knockdown was also diminished in *hlh-30(tm1978)* mutant, suggesting that *hlh-30* may contribute to *pitp-1*-mediated lifespan regulation (Fig. 5J and Supplementary Table S4). In addition, RNAi knockdown of *skn-1* strongly suppressed, whereas *hlh-30* knockdown partially suppressed, the extended lifespan in *pitp-1* mutants (Supplementary Fig. 4F and 4G), supporting that *skn-1* and *hlh-30* participate in *pitp-1* reduction-mediated longevity. Together, these results suggest that although *pitp-1* is transcriptionally repressed by DAF-16, its pro-longevity effect is not mediated through DAF-16 itself. Instead, *pitp-1* negatively modulates lifespan through coordinated regulation of the AKT–TOR signaling axis and downstream transcriptional regulators, including SKN-1 and HLH-30.

### Transcriptome-wide analyses uncover signaling shifts and longevity mechanisms upon *pitp-1* suppression

**Fig. 6:**
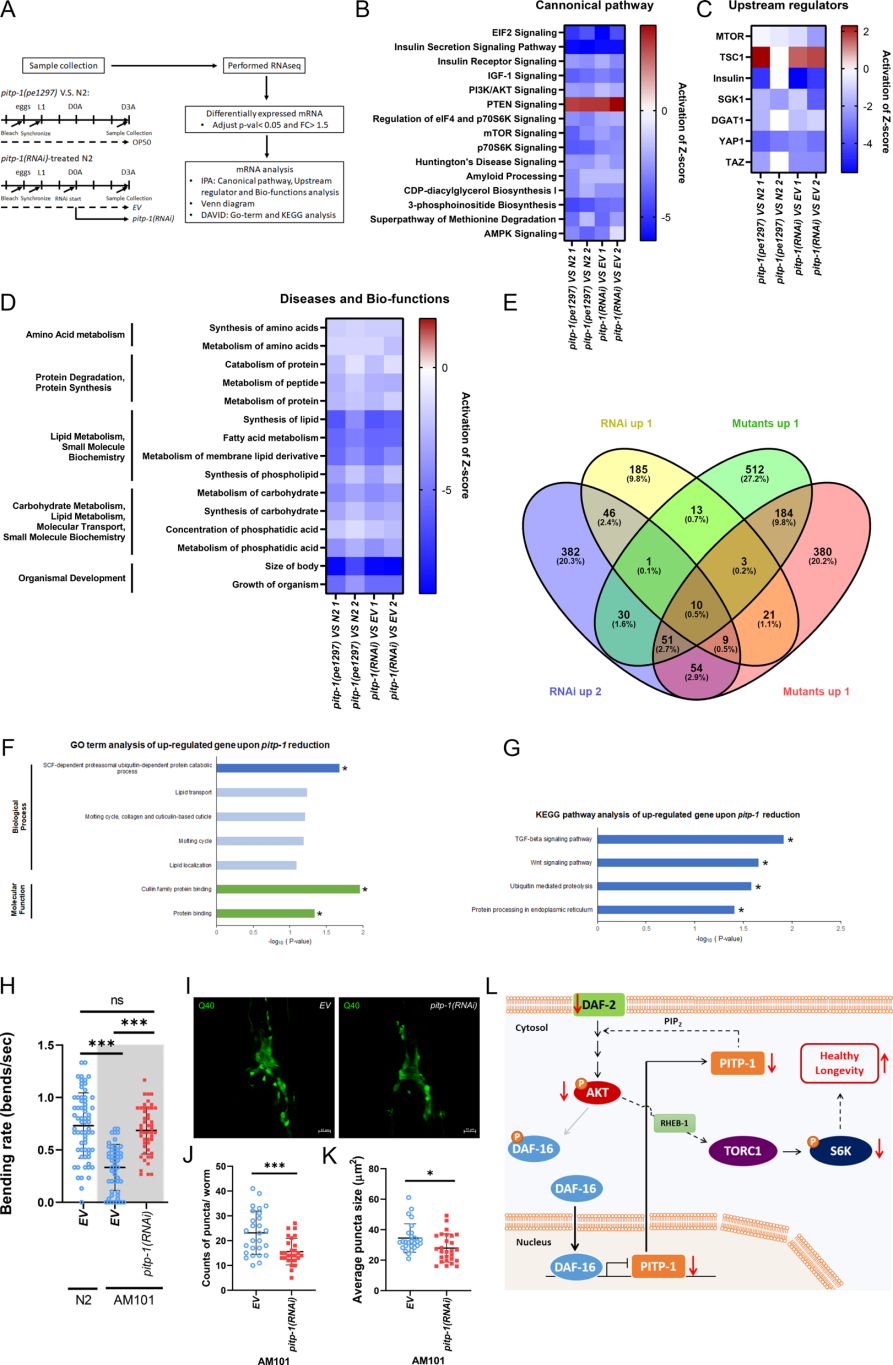
Transcriptomic and pathway analysis upon *pitp-1* reduction. RNA-seq and pathway enrichment analyses revealed downregulation of insulin/TOR signaling and upregulation of proteolysis-related genes in *pitp-1* mutants and RNAi-treated worms. **A** Schematic diagram of RNA-seq samples collection and data analysis. **B** The results of IPA canonical pathway analysis upon *pitp-1* reduction. **C** The results of IPA upstream regulator analysis upon *pitp-1* reduction. **D** The results of IPA disease and biofunctions analysis upon *pitp-1* reduction. **E** ORA analysis identified potential up-regulated downstream target genes which might be involve in *pitp-1* reduction-mediated longevity. **F** GO terms enrichment analysis of the up-regulated genes upon *pitp-1* reduction (FC > 1.5; adjusted p < 0.05*). The vertical coordinates were the enriched GO terms, and the horizontal coordinates were the numbers of the up-regulated genes in these GO terms. The blue columns represent the biological process GO terms. The green columns represent the molecular function GO terms. GO terms enrichment analysis was conducted by DAVID. **G** KEGG enrichment analysis of the changed genes upon *pitp-1* reduction (FC > 1.5; adjusted p < 0.05*). KEGG enrichment analysis was conducted by DAVID. **H** Knockdown of *pitp-1* improved motility in the AM101 worms expressing neuronal polyQ (Q40::YFP). Data are presented as mean ± SD (n = 3 independent experiments). Statistical significance was determined by One-way ANOVA. **I** Representative fluorescence images of polyQ aggregates in head neurons of AM101 worms under *EV* and *pitp-1(RNAi)* conditions. **J**, **K** Quantification of polyQ aggregation showed reduced puncta number and size upon *pitp-1* knockdown. Each data point represents an individual animal. Data are presented as mean ± SD (n = 3 independent experiments). Statistical significance was determined by Student’s t-test. **L** The working model for *pitp-1* reduction-mediated lifespan regulation in *C. elegans*

Since the longevity effect of *pitp-1* suppression is temporally restricted, and likely involves complex transcriptional reprogramming and pathway cross-talk, we performed RNA sequencing on D3A worms with reduced *pitp-1* expression to gain a comprehensive understanding of transcriptomic changes. Transcriptomic profiles were analyzed using Ingenuity Pathway Analysis (IPA) and Over-Representation Analysis (ORA) (Fig. 6A). IPA canonical pathway analysis revealed downregulation of IIS, PI3K/AKT, and TOR in *pitp-1* mutant and *pitp-1(RNAi)*-treated worms (Fig. 6B), consistent with our mechanism study findings. In addition, PTEN signaling, the negative regulator of IIS, was activated upon *pitp-1* suppression, supporting that *pitp-1* reduction leads to attenuation of IIS. In contrast, AMPK signaling was not activated, which is consistent with our previous results showing that *pitp-1* reduction does not promote longevity through AMPK activation (Supplementary Fig. 3L). Similarly, IPA upstream regulator analysis suggests that the transcriptomic changes we observed may result from decreased upstream activity of mTOR or insulin signaling (Fig. 6C). Together, these results reinforce the critical roles of IIS and TOR signaling in mediating *pitp-1*-dependent longevity.

In addition to IIS and TOR, eIF2 signaling was also significantly downregulated in *pitp-1*-reduced worms (Fig. 6B). As a key regulator of translation initiation, inhibition of eIF2B enhances proteostasis and extends lifespan [35, 49, 50]. Consistently, *pitp-1* suppression reduced protein synthesis (Fig. 4G–J), accompanied by extended lifespan. Together, these observations suggest that *pitp-1* reduction promotes longevity, perhaps by improving protein homeostasis.

We also found that *pitp-1* suppression downregulated CDP-DAG biosynthesis pathway and 3-phosphoinositide biosynthesis (Fig. 6B), suggesting PIP cycle activity may be downregulated. Consistently, IPA Diseases and Bio-functions analysis (Fig. 6D) revealed decreased lipid metabolism, amino acid metabolism, and protein synthesis, likely reflecting TOR suppression. This metabolic reduction may also explain the downregulation of biofunctions such as "size of body" and "growth of organism" (Fig. 6D). These results align with our earlier observation that *pitp-1* mutants exhibit smaller body size (Supplementary Fig. 1E).

In addition to IPA, we employed ORA to identify downstream gene targets upon *pitp-1* reduction. Using a cutoff of > 1.5-fold change and adjusted *p*-value < 0.05, we identified 10 genes significantly upregulated upon *pitp-1* suppression (Fig. 6E). GO and KEGG pathway analyses revealed that the “SCF-dependent, ubiquitin-mediated proteasomal protein catabolic process”, “ubiquitin-mediated proteolysis” and “Protein processing in endoplasmic reticulum” were significantly overrepresented (Fig. 6F, G). This result aligns with our IPA analysis showing reduced global protein synthesis (Fig. 6D) and our puromycin incorporation assay (Fig. 4G–J). Given that enhancing proteolytic systems, including the ubiquitin–proteasome pathway, promotes longevity [51, 52], our findings suggest that *pitp-1* reduction may promote healthy longevity by maintaining proteostasis.

Impaired proteostasis is a hallmark of aging and is often characterized by the accumulation of aggregation-prone proteins, such as polyQ-containing proteins [53]. Accordingly, polyQ aggregation models in *C. elegans* are widely used as a functional assay to evaluate organismal proteostasis capacity [32, 53]. To further assess whether *pitp-1* reduction improves proteostasis, we utilized the AM101 strain expressing neuronal polyQ (Q40::YFP). Knockdown of *pitp-1* significantly improved motility in AM101 worms, as evidenced by an increased bending rate compared to the control group (Fig. 6H). Consistently, knockdown of *pitp-1* reduced polyQ aggregation, as indicated by a significant decrease in puncta number (Fig. 6I and J) and a reduction in puncta size (Fig. 6I and K). These results suggest that reduction of *pitp-1* ameliorates proteotoxic stress and improves proteostasis in a polyQ aggregation model.

In summary, these transcriptomic analyses reinforce that *pitp-1* reduction promotes longevity through coordinated suppression of IIS, TOR signaling, reducing anabolic activity, and enhancing proteostasis. Our data support a model in which DAF-16 represses *pitp-1* transcription under reduced IIS, partially contributing to IIS-mediated longevity. Reduced *pitp-1* attenuates TOR activity via the AKT–RHEB axis, thereby promoting longevity. This is the first study to identify *pitp-1* as a novel lifespan regulator and highlights its involvement in IIS–TOR crosstalk, offering new insights into aging regulation and potential anti-aging interventions.

## Discussion

PITP is a critical regulator in the PIP cycle, but its role in aging remains unclear. In this study, we identify a previously unrecognized function for *pitp-1*, a class II PITP, in regulating lifespan and healthspan in *C. elegans*. We found that *pitp-1* is transcriptionally repressed by DAF-16 and acts as a pro-aging factor, at least in part through modulation of TOR signaling. Notably, our spatial and temporal analyses reveal that both neuronal specificity and early adulthood timing are essential for *pitp-1*-mediated lifespan regulation. In addition, our genetic analyses indicate that SKN-1 and HLH-30, another two key downstream transcription factors of IIS, also contribute to *pitp-1*-mediated lifespan regulation. These findings position *pitp-1* as a regulator linking IIS and TOR signaling in aging control, with its pro-aging function constrained by specific neuronal and temporal contexts.

Our temporal analysis highlighted a critical window during early adulthood, particularly the early reproductive stages, as essential for *pitp-1*-mediated lifespan regulation, with adult-onset knockdown sufficient to promote healthy longevity without interfering with development. This time window is consistent with the concept that interventions in nutrient-sensing pathways are most effective during this stage [36–38]. These parallels highlight that *pitp-1* reduction aligns with these conserved longevity-regulating pathways during a critical early-adult temporal window. Moreover, our analysis of public gene expression datasets disclosed a natural, age-associated decline in *pitp-1/PITPNMs* expression in worms and humans (Supplementary Fig. 2) [39, 40], suggesting that this downregulation may represent a conserved protective mechanism against aging.

Spatially, neuronal knockdown of *pitp-1* is sufficient to promote longevity, emphasizing the central role of the nervous system in systemic aging regulation where IIS and TOR signaling exert their lifespan-modulating effects [41, 42, 54]. Consistent with this, *pitp-1* has been reported to be predominantly expressed in neurons in the head region, particularly in sensory neurons such as ASE and AWC, suggesting a potential role in neuronal signaling processes relevant to aging [18]. In our study, *pitp-1*::GFP expression was also primarily observed in head neurons, although we did not perform colocalization analysis to define specific neuronal subtypes. Notably, lifespan assays using feeding RNAi in wild-type animals may reflect contributions from multiple tissues. Together with neuron-restricted RNAi results, these findings suggest that *pitp-1* functions in neurons to regulate lifespan, while its modulation through RNAi may involve both neuronal and non-neuronal components depending on the experimental context. Consistently, neuronal inhibition of RAGA-1 from hatching or D1A extends lifespan, supporting the temporal flexibility of neuronal TOR suppression in promoting longevity [55]. In addition, other PIP cycle genes, including *dagl-1* in *C. elegans* and its *D. melanogaster* ortholog *inaE*, as well as *PLCβ* homolog *egl-8* in *C. elegans*, expressed in neurons also regulate lifespan through the TOR pathway [7, 56]. These findings underline that neuronal modulation of PI signaling impacts systemic aging via TOR, in line with the role we propose for *pitp-1*.

Moreover, suppression of *pitp-1* in either glutamatergic, cholinergic, or GABAergic neurons each extended lifespan, suggesting that *pitp-1* exerts its pro-aging function through multiple excitatory and inhibitory neuronal circuits. Inhibition of age-related increases in neural excitation, particularly in glutamatergic and cholinergic neurons, extends lifespan [57]. Chronic hyperexcitability of glutamatergic neurons accelerates aging by PLCβ–IP3R pathway overactivation, and suppressing this pathway restores normal lifespan [57, 58]. Additionally, mTOR hyperactivation enhances synaptic responses in glutamatergic and GABAergic neurons, while rapamycin treatment normalizes glutamatergic overexcitation and restores neurotransmitter balance [59]. These data suggest that PLCβ–IP3R pathway or mTOR suppression in excitatory and inhibitory neurons promotes neural homeostasis and healthy aging, aligning with our findings. Taken together, *pitp-1* may function as a neuronal regulator of longevity acting through TOR, potentially by modulating neuronal excitability and neurotransmission. Future studies should clarify the role of distinct circuits and how *pitp-1* coordinates PIP cycle to systemic metabolic responses upon aging.

eIF2 is a central regulator of translation initiation, whose activity is inhibited by phosphorylation of eIF2α, leading to global translational repression and proteostasis maintenance [35, 49, 50]. Our IPA analysis indicated that *pitp-1* downregulation decreases eIF2 signaling, consistent with reduced global translation. Moreover, prior studies have reported bidirectional crosstalk between eIF2 and mTORC1. mTORC1 inhibition can activate GCN2 to phosphorylate eIF2α, whereas eIF2α phosphorylation and ATF4 translation can inhibit mTORC1 by REDD1 and Sestrin2 induction [60–62]. In line with this, we found that *pitp-1* downregulation not only represses TOR signaling but also requires *sestrin* for lifespan extension (Supplementary Fig. 3 M, 3N), suggesting the involvement of the eIF2α–ATF4–Sestrin axis. This pathway acts independently of AMPK [60], consistent with our data (Supplementary Fig. 3L), and may inhibit TOR by restraining Rag GTPase–mediated activation [43]. Together, our findings suggest that the observed downregulation of eIF2 signaling upon *pitp-1* reduction may potentially contribute to TOR inhibition and healthy longevity, while further studies are needed to establish a direct causal role in the future.

In this study, we also found that *pitp-1* suppression in *C. elegans* not only promotes longevity but also results in reduced body size, a phenotype often linked to altered nutrient signaling. In addition to reduced TOR or IIS activity, the two nutrient-sensing pathways known to influence body size, our transcriptomic analysis further revealed a downregulation of YAP and TAZ in *pitp-1*-suppressed worms, as predicted by upstream regulator analysis using IPA (Fig. 6C). This finding is consistent with recent studies in mammalian systems showing that inhibition of PITPα/β activates the Hippo pathway, leading to suppression of YAP-mediated transcription, reduced cell proliferation, and enhanced cancer cell death [61]. Our observation of reduced YAP/TAZ activity and smaller body size upon *pitp-1* suppression may reflect a conserved mechanism, where diminished PI4P-mediated suppression of the Hippo pathway contributes to reduced growth. Importantly, these findings raise the possibility that modulation of the Hippo pathway or direct regulation of its downstream transcription factors YAP/TAZ may represent a potential strategy to promote healthy aging. In particular, investigating how *pitp-1* interfaces with the Hippo pathway may uncover a previously unrecognized lipid-signaling mechanism with relevance to both growth regulation and age-associated functional decline.

In addition to downregulation of canonical nutrient-sensing pathways, eIF2 signaling and Hippo pathway, our IPA analysis revealed a suppression of Huntington’s disease (HD) signaling upon *pitp-1* reduction (Fig. 6B). Given that mutated Huntingtin (Htt) enhances mTORC1 activity through Rheb interaction and aberrant PI3K/AKT/mTOR signaling contributes to HD pathogenesis [63, 64], these findings are consistent with our model that *pitp-1* modulates lifespan through Rheb-TOR signaling and shows downregulation of HD-associated signaling. Impaired proteostasis, characterized by the accumulation of aggregation-prone proteins such as polyQ-containing proteins, is a key feature of HD and aging. Consistent with the transcriptomic prediction, we found that *pitp-1* knockdown alleviated polyQ proteotoxicity in the neuronal AM101 model, as evidenced by improved motility and reduced aggregation burden, including decreased puncta number and size (Fig. 6H–K). These findings provide functional evidence that *pitp-1* reduction enhances organismal proteostasis, supporting a role for *pitp-1* reduction in alleviating protein aggregation–associated toxicity. Notably, HD is characterized by dysfunction of both GABAergic and glutamatergic neurons, which are central to motor impairment and excitotoxicity [65–67]. Intriguingly, suppression of *pitp-1* in either GABAergic or glutamatergic neurons was sufficient to extend lifespan in *C. elegans* (Fig. 2), suggesting that *pitp-1* may act through conserved neuronal circuits also implicated in HD pathogenesis. Together, these findings raise the possibility that *pitp-1* suppression may contribute to promoting healthy longevity and mitigating proteotoxic stress associated with neurodegenerative diseases such as HD. Future investigation exploring whether *pitp-1/Nir2* modulation can mitigate HD-related phenotypes in mammalian systems will help to evaluate its therapeutic potential.

Consistently, our parallel study in *D. melanogaster* revealed that downregulation of *rdgB*, the orthologue of *pitp-1*, also promotes healthy longevity and reduces TOR activity (data not shown). These findings suggest that the pro-aging role of PITPs and their regulation of TOR signaling could be evolutionarily conserved. Thus, the mechanisms uncovered here may extend beyond nematodes and flies, raising the exciting possibility that PITP modulation could exert similar effects on aging and healthspan in mammals, including humans.

While our genetic and biochemical data consistently support a role for PITP-1 in modulating TOR signaling, with additional involvement of IIS, the precise molecular mechanism directly linking PITP-1 to these pathways remains to be elucidated. The consistent phenotypes observed across independent mutant alleles and RNAi-mediated knockdown worms strongly support that reduced *pitp-1* expression underlies the observed lifespan regulation. Future studies using catalytically inactive mutants and rescue approaches will help to determine whether the lipid transfer activity of PITP-1 is essential for these effects. In addition, RNAi efficiency may vary depending on gene targets and tissue specificity. Therefore, in addition to the direct neuronal effect, cell non-autonomous mechanisms may also contribute to the regulation of *pitp-1* expression and its impact on lifespan. Notably, the *pitp-1* promoter activity assays were performed in a wild-type background, where neuronal RNAi is limited. Thus, the observed regulation of *pitp-1* expression by IIS components (*daf-2* and *daf-16*) may reflect systemic, cell non-autonomous effects rather than direct neuronal regulation. In addition, RNAi-based results, especially negative findings, should be interpreted with caution.

Taken together, despite these remaining questions, our genetic and functional evidence consistently supports a model in which PITP-1 functions as a previously unrecognized lifespan regulator through modulation of IIS and TOR signaling, providing new insights into how nutrient-sensing pathways are coordinated to control lifespan.

## Conclusions

Our findings uncover *pitp-1* as a new regulator of aging linking IIS and TOR signaling in a neuron- and age-specific manner. This study supports a role for *pitp-1* as a critical node coordinating nutrient-sensing pathways to regulate healthy longevity and highlights its potential as a target for aging-associated interventions. While our results reveal its role in IIS-TOR cross talk, they also suggest that *pitp-1* may influence additional pathways implicated in growth control, proteostasis, and neurodegeneration, including Hippo, eIF2, and Huntington’s disease–related signaling. These findings warrant further investigation, particularly in other species such as *D. melanogaster* or mammals, to assess the conservation of this regulatory axis and its relevance to healthy aging and neurodegenerative diseases.

## Supplementary Information

Supplementary material 1. Supplementary Fig. 1. The reduction of class II PITP, *pitp-1*, reveals longevity-related phenotypes. (A) Schematic diagram of the *pitp-1* genomic locus and mutant alleles. The *pitp-1(pe1297)* allele carries an approximately 2.95 kb genomic deletion replaced by a ~ 2.2 kb *C. briggsae unc-119(* +*)* cassette, and has been described as a candidate null allele. The *pitp-1(tm1500)* allele contains a deletion spanning coding regions and is predicted to cause a frameshift and premature truncation. (B) Schematic representation of the PITP-1 protein domain structure and predicted effects of the *pitp-1* mutant alleles. PITP-1 contains an N-terminal PITP domain, followed by DDHD and LNS2 domains. The *pitp-1(pe1297)* allele is predicted to result in near-complete loss of the PITP domain (candidate null allele), whereas *pitp-1(tm1500)* is predicted to produce a truncated protein retaining only the N-terminal PITP domain. (C) Knockdown of *pitp-1*, but not other class I PITP homologs, extended lifespan in N2. (D-F) qPCR confirmed RNAi targeting class I PITP homologs specifically reduced their own transcript levels without affecting *pitp-1*. (G) *pitp-1* mutants exhibited reduced body size. Each data point represents an individual animal. Data are presented as mean ± SD (n = 3 independent experiments). (H) Schematic diagram of RNAi treatment timelines. (I) *pitp-1* knockdown during the reproductive stage promotes longevity. Survival curves are representative of three independent biological replicates. Data are presented as mean ± SD (n = 3 independent experiments) for quantitative analyses. Statistical significance was determined by log-rank test for lifespan assays, ANOVA for multiple comparisons.

Supplementary material 2. Supplementary Fig. 2. GEO shows reduced class II PITP expression in old age. (A) Whole-genome microarray data from *C. elegans* [39] revealed significant reductions in both *pitp-1* splice variants at day-6 and day-15 adults compared to L4 larvae (One-way ANOVA). (B-G) Microarray analysis of human frontal cortex [40] showed that expression of PITPNM2 (232950_at) and PITPNM3 (230076_at) was significantly lower in individuals > 90 years (extremely old) compared to those < 40 years (young) in both sexes, while PITPNM1 showed a slight, non-significant decrease (unpaired Student’s t-test).

Supplementary material 3. Supplementary Fig. 3. *pitp-1* negatively regulates lifespan by modulating TOR signaling. (A–B) RNAi knockdown of *pitp-1* did not further prolong the extended lifespan in two *dgk-5* mutants. (C, D) The elevated p-S6K levels in PITP-1 overexpression strains were reverted by genetic inhibition of TOR signaling (*let-363*, *raga-1*). (E, F) The reduced p-S6K levels in two *pitp-1* mutants were reverted by PITP-1 overexpression. (G, H) Genetic or pharmacological inhibition of TOR did not further enhance the extended lifespan in *pitp-1(tm1500)* mutant. (I) Knockdown of *pitp-1* did not further prolong the enhanced lifespan in *rsks-1* mutants. (J) Genetic knockdown of TOR by *let-363(RNAi)* rescued the motility decline caused by PITP-1 overexpression. (K) Schematic diagram of TOR upstream regulators RAG, RHEB, AMPK. (L) Knockdown of *pitp-1* extended lifespan in *aak-2(gt33)*. (M, N) *sesn-1* mutation blocked the longevity effect of *pitp-1(RNAi)* knockdown. Survival curves are representative of at least two independent experiments (n = 2–3 depending on the strain). Data are presented as mean ± SD (n = 3—4 independent experiments) for quantitative analyses. Statistical significance was determined by log-rank test for lifespan assays, ANOVA for multiple comparisons.

Supplementary material 4. Supplementary Fig. 4. The role of *pitp-1* in IIS-mediated lifespan regulation. (A) Knockdown of *pitp-1* did not promote DAF-16 nuclear translocation. TJ356[*daf-16p::daf-16a/b::GFP* + *rol-6(su1006)*] was used as a DAF-16 reporter strain. Red arrows indicated DAF-16::GFP translocated into the nucleus and forms GFP puncta by *daf-2(RNAi)* as the positive control. (B, C) Knockdown of *pitp-1* did not increase *sod-3* expression. CF1553[*sod-3p::GFP* + *rol-6(su1006)*] was used as a *sod-3* reporter strain. (D) Whole-genome microarray data from *C. elegans* [45] revealed *pitp-1* expression was significantly reduced in *daf-2(e1370)*. (E) PITP-1 overexpression partially blocked the longevity effect by *age-1(RNAi)* knockdown. Survival curves are representative of three independent experiments. Data are presented as mean ± SD (n = 3 biological replicates) for quantitative analyses. Statistical significance was determined by log-rank test for lifespan assays, ANOVA for multiple comparisons, and unpaired Student’s t-test where applicable.

Supplementary material 5.

Supplementary material 6.

Supplementary material 7.

Supplementary material 8.

Supplementary material 9.

## Data Availability

All the RNAseq raw data can be accessed by the GEO accession number GSE309580.

## Abbreviations

pitp-1: Phosphatidylinositol transfer protein-1
PITPs: Phosphatidylinositol transfer proteins
*C. elegans*: *Caenorhabditis elegans*
*D. melanogaster*: *Drosophila melanogaster*
PI: Phosphatidylinositol
PA: Phosphatidic acid
ER: Endoplasmic reticulum
PM: Plasma membrane
PIP: Phosphoinositide
PIP_2_: Phosphatidylinositol 4,5-bisphosphate
DAG: Diacylglycerol
IIS: Insulin/IGF-1 signaling
TOR: Target of rapamycin
p-S6K: Phosphorylated S6K
p-AKT: Phosphorylated AKT
TORC1: TOR complex 1
TORC2: TOR complex 2
PLCβ: Phospholipase C β
EV: Empty vector
NC membrane: Nitrocellulose (NC) membrane
GEO: Gene Expression Omnibus
RNA-seq: RNA sequencing
RIN: RNA Integrity Number
IPA: Ingenuity Pathway Analysis
ORA: Over-representation analysis
GO: Gene ontology
CGC: Caenorhabditis Genetics Center
NBRP: National BioResource Project
NGM: Nematode growth medium
FUdR: 5-Fluoro-2’-deoxyuridine
DMSO: Dimethyl sulfoxide
Juglone: 5-Hydroxyl-1,4-naphthoquinone
DTT: Dithiothreitol
polyQ: Polyglutamine
NSTC: National Science and Technology Council
