# Supplementary figures and images for: Phosphatidylinositol transfer protein-1 integrates insulin/IGF-1 and TOR signaling to negatively regulate lifespan and healthspan in Caenorhabditis elegans
Source: J Biomed Sci. 2026 Apr 27;33:42. doi: 10.1186/s12929-026-01246-x (PMC13112645; doi:10.1186/s12929-026-01246-x)

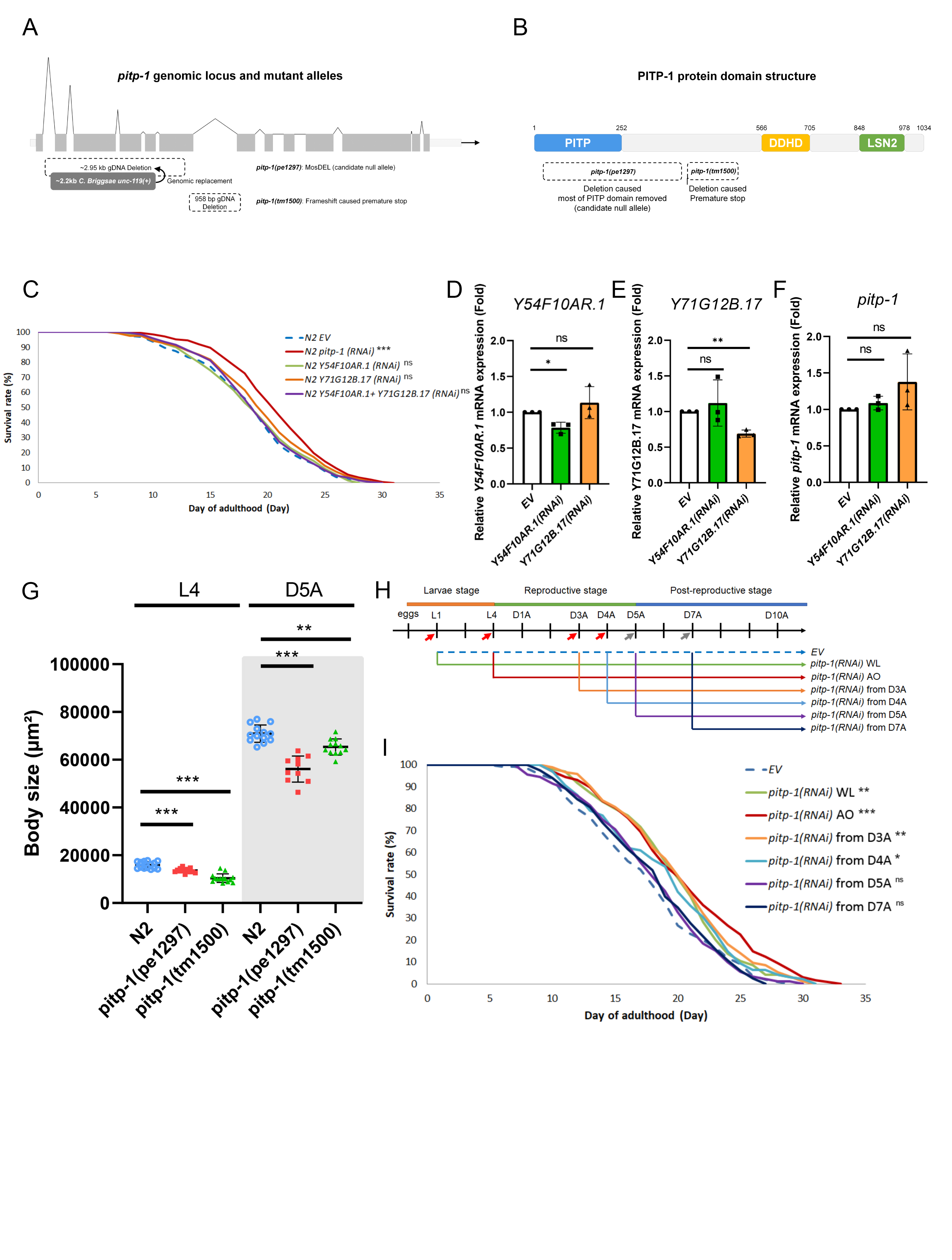

Supplement: Supplementary file 1 — Supplementary material 1. Supplementary Fig. 1. The reduction of class II PITP, pitp-1, reveals longevity-related phenotypes. (A) Schematic diagram of the pitp-1 genomic locus and mutant alleles. The pitp-1(pe1297) allele carries an approximately 2.95 kb genomic deletion replaced by a ~ 2.2 kb C. briggsae unc-119( +) cassette, and has been described as a candidate null allele. The pitp-1(tm1500) allele contains a deletion spanning coding regions and is predicted to cause a frameshift and premature truncation. (B) Schematic representation of the PITP-1 protein domain structure and predicted effects of the pitp-1 mutant alleles. PITP-1 contains an N-terminal PITP domain, followed by DDHD and LNS2 domains. The pitp-1(pe1297) allele is predicted to result in near-complete loss of the PITP domain (candidate null allele), whereas pitp-1(tm1500) is predicted to produce a truncated protein retaining only the N-terminal PITP domain. (C) Knockdown of pitp-1, but not other class I PITP homologs, extended lifespan in N2. (D-F) qPCR confirmed RNAi targeting class I PITP homologs specifically reduced their own transcript levels without affecting pitp-1. (G) pitp-1 mutants exhibited reduced body size. Each data point represents an individual animal. Data are presented as mean ± SD (n = 3 independent experiments). (H) Schematic diagram of RNAi treatment timelines. (I) pitp-1 knockdown during the reproductive stage promotes longevity. Survival curves are representative of three independent biological replicates. Data are presented as mean ± SD (n = 3 independent experiments) for quantitative analyses. Statistical significance was determined by log-rank test for lifespan assays, ANOVA for multiple comparisons. [file 12929_2026_1246_MOESM1_ESM.tif]

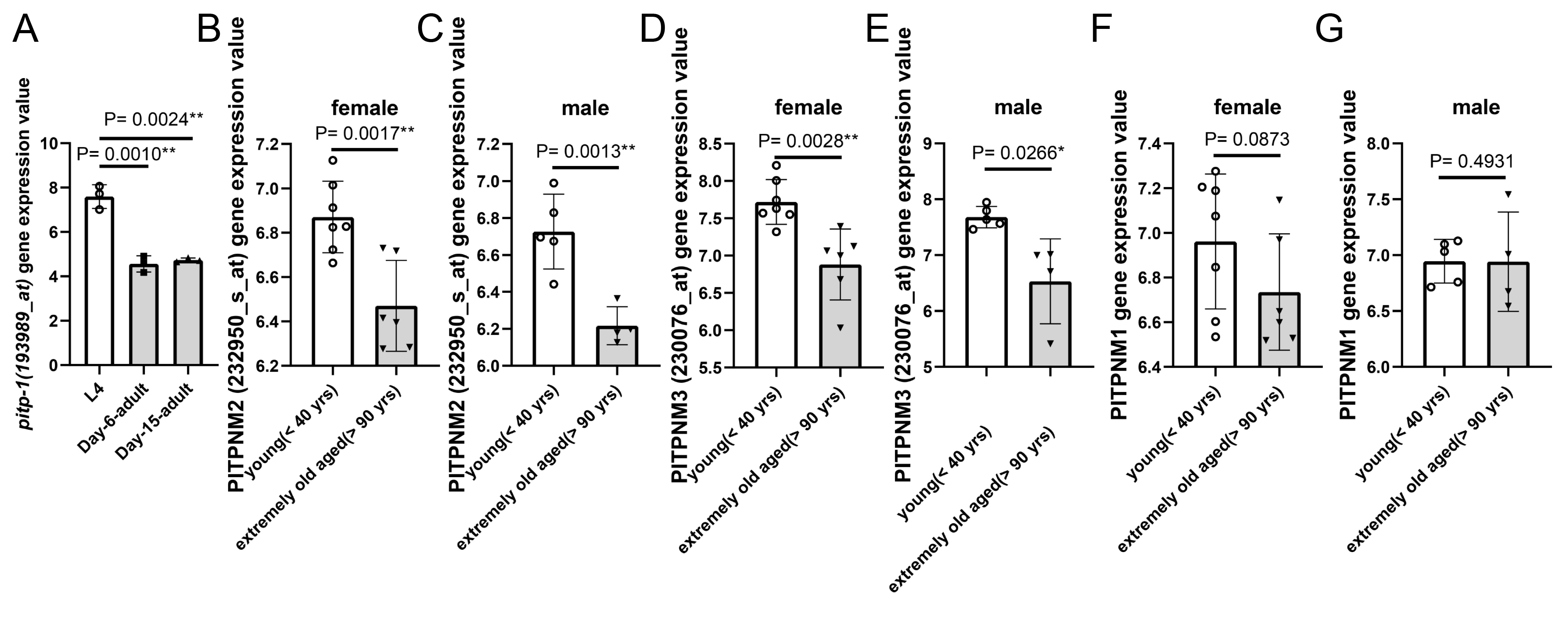

Supplement: Supplementary file 2 — Supplementary material 2. Supplementary Fig. 2. GEO shows reduced class II PITP expression in old age. (A) Whole-genome microarray data from C. elegans [39] revealed significant reductions in both pitp-1 splice variants at day-6 and day-15 adults compared to L4 larvae (One-way ANOVA). (B-G) Microarray analysis of human frontal cortex [40] showed that expression of PITPNM2 (232950_at) and PITPNM3 (230076_at) was significantly lower in individuals > 90 years (extremely old) compared to those < 40 years (young) in both sexes, while PITPNM1 showed a slight, non-significant decrease (unpaired Student’s t-test). [file 12929_2026_1246_MOESM2_ESM.tif]

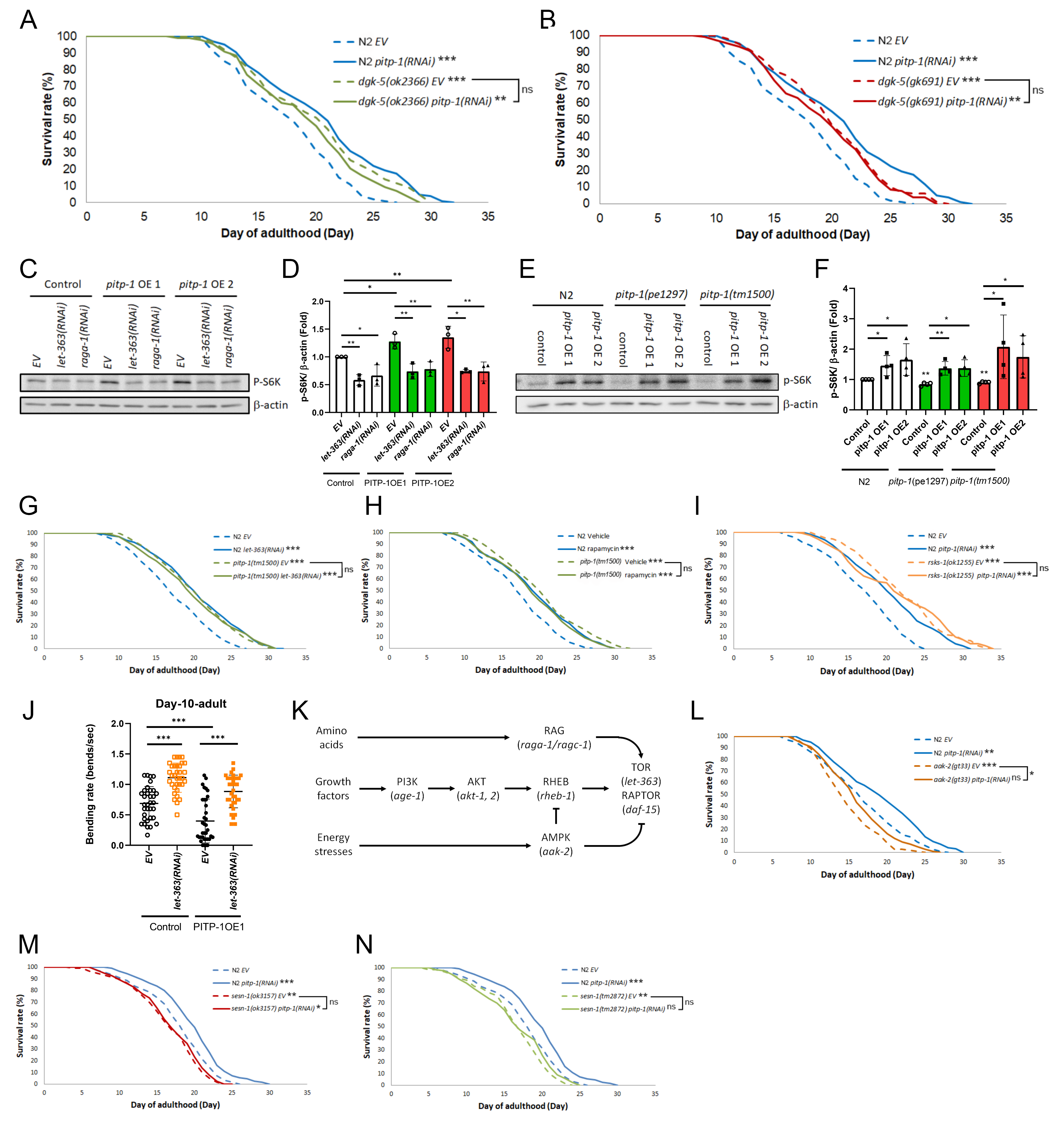

Supplement: Supplementary file 3 — Supplementary material 3. Supplementary Fig. 3. pitp-1 negatively regulates lifespan by modulating TOR signaling. (A–B) RNAi knockdown of pitp-1 did not further prolong the extended lifespan in two dgk-5 mutants. (C, D) The elevated p-S6K levels in PITP-1 overexpression strains were reverted by genetic inhibition of TOR signaling (let-363, raga-1). (E, F) The reduced p-S6K levels in two pitp-1 mutants were reverted by PITP-1 overexpression. (G, H) Genetic or pharmacological inhibition of TOR did not further enhance the extended lifespan in pitp-1(tm1500) mutant. (I) Knockdown of pitp-1 did not further prolong the enhanced lifespan in rsks-1 mutants. (J) Genetic knockdown of TOR by let-363(RNAi) rescued the motility decline caused by PITP-1 overexpression. (K) Schematic diagram of TOR upstream regulators RAG, RHEB, AMPK. (L) Knockdown of pitp-1 extended lifespan in aak-2(gt33). (M, N) sesn-1 mutation blocked the longevity effect of pitp-1(RNAi) knockdown. Survival curves are representative of at least two independent experiments (n = 2–3 depending on the strain). Data are presented as mean ± SD (n = 3—4 independent experiments) for quantitative analyses. Statistical significance was determined by log-rank test for lifespan assays, ANOVA for multiple comparisons. [file 12929_2026_1246_MOESM3_ESM.tif]

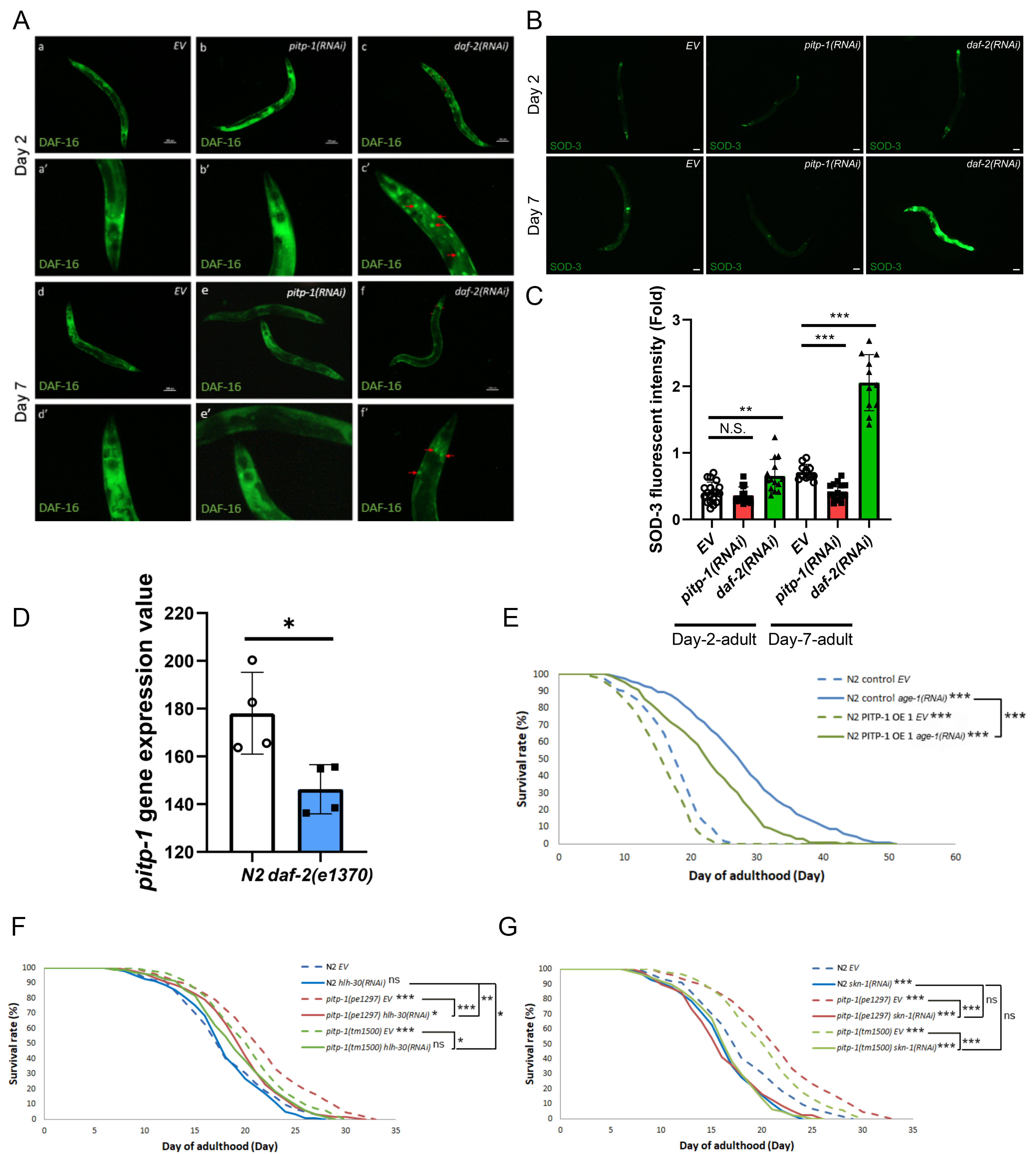

Supplement: Supplementary file 4 — Supplementary material 4. Supplementary Fig. 4. The role of pitp-1 in IIS-mediated lifespan regulation. (A) Knockdown of pitp-1 did not promote DAF-16 nuclear translocation. TJ356[daf-16p::daf-16a/b::GFP + rol-6(su1006)] was used as a DAF-16 reporter strain. Red arrows indicated DAF-16::GFP translocated into the nucleus and forms GFP puncta by daf-2(RNAi) as the positive control. (B, C) Knockdown of pitp-1 did not increase sod-3 expression. CF1553[sod-3p::GFP + rol-6(su1006)] was used as a sod-3 reporter strain. (D) Whole-genome microarray data from C. elegans [45] revealed pitp-1 expression was significantly reduced in daf-2(e1370). (E) PITP-1 overexpression partially blocked the longevity effect by age-1(RNAi) knockdown. Survival curves are representative of three independent experiments. Data are presented as mean ± SD (n = 3 biological replicates) for quantitative analyses. Statistical significance was determined by log-rank test for lifespan assays, ANOVA for multiple comparisons, and unpaired Student’s t-test where applicable. [file 12929_2026_1246_MOESM4_ESM.tif]
